# Identification of circulating microRNA profiles associated with pulmonary function and radiologic features in survivors of SARS-CoV-2-induced ARDS

**DOI:** 10.1080/22221751.2022.2081615

**Published:** 2022-06-04

**Authors:** María C. García-Hidalgo, Jessica González, Iván D. Benítez, Paola Carmona, Sally Santisteve, Manel Pérez-Pons, Anna Moncusí-Moix, Clara Gort-Paniello, Fátima Rodríguez-Jara, Marta Molinero, Thalia Belmonte, Gerard Torres, Gonzalo Labarca, Estefania Nova-Lamperti, Jesús Caballero, Jesús F. Bermejo-Martin, Adrián Ceccato, Laia Fernández-Barat, Ricard Ferrer, Dario Garcia-Gasulla, Rosario Menéndez, Ana Motos, Oscar Peñuelas, Jordi Riera, Antoni Torres, Ferran Barbé, David de Gonzalo-Calvo, on behalf of the CIBERESUCICOVID Project (COV20/00110, ISCIII)

**Affiliations:** a Translational Research in Respiratory Medicine, University Hospital Arnau de Vilanova and Santa Maria, IRBLleida, Lleida, Spain; b CIBER of Respiratory Diseases (CIBERES), Institute of Health Carlos III, Madrid, Spain; c Molecular and Translational Immunology Laboratory, Department of Clinical Biochemistry and Immunology, Faculty of Pharmacy, Universidad de Concepcion, Concepcion, Chile; d Internal Medicine Unit, Complejo Asistencial Dr. Víctor Ríos Ruiz, Los Ángeles, Chile; e Grup de Recerca Medicina Intensiva, Intensive Care Department Hospital Universitari Arnau de Vilanova, IRBLleida, Lleida, Spain; f Hospital Universitario Río Hortega de Valladolid, Valladolid, Spain; Instituto de Investigación Biomédica de Salamanca (IBSAL), Salamanca, Spain; g Servei de Pneumologia, Hospital Clinic, Universitat de Barcelona; IDIBAPS, Barcelona, Spain; h Intensive Care Department, Vall d’Hebron Hospital Universitari. SODIR Research Group, Vall d’Hebron Institut de Recerca (VHIR), Barcelona, Spain; i Barcelona Supercomputing Center (BSC), Barcelona, Spain; j Pulmonology Service, University and Polytechnic Hospital La Fe, Valencia, Spain; k Hospital Universitario de Getafe, Madrid, Spain; l Pneumology Department, Clinic Institute of Thorax (ICT), Hospital Clinic of Barcelona - Insitut d'Investigacions Biomèdiques August Pi i Sunyer (IDIBAPS) - ICREA, University of Barcelona (UB), Barcelona, Spain

**Keywords:** Acute respiratory distress syndrome, COVID-19, lung function, microRNA, sequelae, total severity score

## Abstract

There is a limited understanding of the pathophysiology of postacute pulmonary sequelae in severe COVID-19. The aim of current study was to define the circulating microRNA (miRNA) profiles associated with pulmonary function and radiologic features in survivors of SARS-CoV-2-induced ARDS. The study included patients who developed ARDS secondary to SARS-CoV-2 infection (n = 167) and a group of infected patients who did not develop ARDS (n = 33). Patients were evaluated 3 months after hospital discharge. The follow-up included a complete pulmonary evaluation and chest computed tomography. Plasma miRNA profiling was performed using RT-qPCR. Random forest was used to construct miRNA signatures associated with lung diffusing capacity for carbon monoxide (D_LCO_) and total severity score (TSS). Kyoto Encyclopedia of Genes and Genomes (KEGG) and Gene Ontology (GO) enrichment analyses were conducted. D_LCO _< 80% predicted was observed in 81.8% of the patients. TSS showed a median [P_25_;P_75_] of 5 [2;8]. The miRNA model associated with D_LCO_ comprised miR-17-5p, miR-27a-3p, miR-126-3p, miR-146a-5p and miR-495-3p. Concerning radiologic features, a miRNA signature composed by miR-9-5p, miR-21-5p, miR-24-3p and miR-221-3p correlated with TSS values. These associations were not observed in the non-ARDS group. KEGG pathway and GO enrichment analyses provided evidence of molecular mechanisms related not only to profibrotic or anti-inflammatory states but also to cell death, immune response, hypoxia, vascularization, coagulation and viral infection. In conclusion, diffusing capacity and radiological features in survivors from SARS-CoV-2-induced ARDS are associated with specific miRNA profiles. These findings provide novel insights into the possible molecular pathways underlying the pathogenesis of pulmonary sequelae.

**Trial registration:** ClinicalTrials.gov identifier: NCT04457505..

**Trial registration:** ISRCTN.org identifier: ISRCTN16865246..

## Introduction

Persistent pulmonary dysfunction has been described as a common clinical feature in survivors of acute respiratory distress syndrome (ARDS) [1,2]. Indeed, 15-50% of patients who recovered from ARDS as a consequence of coronavirus pneumonia, such as in severe acute respiratory syndrome (SARS) and Middle East respiratory syndrome (MERS), experienced major long-term sequelae after hospital discharge (3-24 months), including impaired lung diffusing capacity for carbon monoxide (D_LCO_) and radiological abnormalities [3–5]. Our group recently demonstrated that up to 80% of survivors of ARDS secondary to SARS-CoV-2 infection showed diffusion impairment and pulmonary structural alterations in a 3-month follow-up study [6]. Similar findings have been reported in different cohorts of discharged COVID-19 patients [7–11].

Given the significant burden of pulmonary sequelae caused by SARS-CoV-2 and the current lack of evidence to treat lung dysfunction in “post-COVID” syndrome, the development of innovative therapies is of vital importance. In this scenario, molecular phenotyping of the patient emerges as an essential tool to identify the underlying biological factors implicated in the sequelae and, consequently, targets for intervention [12].

MicroRNAs (miRNAs) are single-stranded noncoding RNA sequences (19-25 nt) that function as posttranscriptional repressors of gene expression. miRNAs participate in complex and coordinated regulatory networks [13], with a single miRNA regulating large sets of genes and a single gene able to be targeted by large sets of miRNAs. This biological system allows the fine-tuning of the cellular phenotype. As such, miRNAs constitute essential modulators of cellular development, homeostasis and response to stress [14]. miRNAs have also been described in the extracellular space [15], including biofluids such as plasma [16]. Extracellular miRNAs could act as signalling molecules at the paracrine and endocrine levels to regulate a broad array of physiological processes [17,18]. Their aberrant expression plays a significant role in the onset and development of pathological conditions, including pulmonary diseases [19,20].

Here, we evaluated the association between pulmonary function and radiologic features and the circulating miRNA profile in survivors of SARS-CoV-2-induced ARDS 3 months after hospital discharge. The final aim is to decipher the pathophysiology of the postacute pulmonary sequelae of severe COVID-19. To the best of our knowledge, this is the first study that uses an approach based on the miRNA transcriptome to characterize the pulmonary function and structural outcomes of ARDS secondary to SARS-CoV-2 infection.

## Methods

### Ethics statements

The study protocol was approved by the medical ethics committee of Hospital Universitari Arnau de Vilanova (CEIC/2273) and the Institutional Review Board (IRB) from Servicio de Salud Bio (IRB: CEC113) and Servicio de Salud Concepcion (IRB: CEC-SSC: 20-07-26). The study protocol was registered in the ISRCTN registry (ID: ISRCTN16865246). The study was performed in full compliance with the Declaration of Helsinki. The patients received written information about the nature and goals of the study and signed an informed consent form. Patient data and sample data are kept in a database with restricted access.

### Study design and population

This is a substudy of the ongoing multicenter study CIBERESUCICOVID registered at www.clinicaltrials.gov with the identification NCT04457505 [21]. This was a prospective study including patients admitted due to severe COVID-19 at Hospital Universitari Arnau de Vilanova-Santa María (Lleida, Spain) between March and December 2020. Patients were evaluated in a “post-COVID” consultation 3 months after hospital discharge in the same hospital (median [P_25_;P_75_] of 96.00 [85.75;107.00] days). The final study population was selected according to the following inclusion criteria: i) positive for SARS-CoV-2 according to a standardized test; ii) aged over 18; iii) developed ARDS according to the Berlin definition [22] during hospital stay; and iv) attended a “post-COVID” consultation. The exclusion criteria for the “Post-COVID” evaluation were: i) less than 1 year of life expectancy; ii) transferred to another institution; iii) treated with palliative care; and iv) impossible to evaluate performance due to severe mental disability. A control group composed by patients positive for SARS-CoV-2 but who did not develop ARDS was additionally included. Patients were evaluated at Hospital Universitari Arnau de Vilanova-Santa María (Lleida, Spain), Hospital Complejo Víctor Ríos Ruiz (Los Ángeles, Chile) and Hospital Guillermo Grant Benavente (Concepción, Chile) between July 2020 and August 2021. Demographic, clinical, pharmacological and laboratory data were abstracted from the electronic medical records and entered into a REDCap database.

### Pulmonary evaluation

A complete pulmonary evaluation was performed as previously described [6]. Airway function was measured using a flow spirometer (MasterScreen, Jaeger, Germany) according to the guidelines of the American Thoracic Society [23]. The results were expressed as a percentage of the D_LCO_ predicted value according to the European Community Lung Health Survey [24]. Chest computed tomography (CT) examinations were performed using a sixteen- and sixty-four-slice multidetector CT scanner (Brilliance 16 and 64; Philips Healthcare). Images were acquired with patients in the supine position in the cranio-caudal direction at end-inspiration. To quantify the severity of lung affectation, the total severity score (TSS) was assessed. Each of the five lung lobes was determined for the percentage of lobar involvement. Following this, the severity of each lobe was classified as none (0%), minimal (1-25%), mild (26-50%), moderate (51-75%), or severe (76-100%), with a corresponding score of 0, 1, 2, 3 or 4, respectively. The TSS is calculated by summing the five lobe scores (range from 0 to 20) [25].

### Samples

Samples collected at the Hospital Universitari Arnau de Vilanova-Santa María (Lleida, Spain) were processed in standardized conditions with support by IRBLleida Biobank (B.0000682) and “Plataforma Biobancos PT20/00021”. Samples collected at the University of Concepción (Concepción, Chile) were processed using standardized conditions according to international Biobank regulations. Venous blood samples were obtained in ethylenediaminetetraacetic acid (EDTA) blood collection tubes (BD, NJ, USA) by venipuncture after a night of fasting and before beginning any interventional procedure at the “post-COVID” consultation 3 months after hospital discharge. To obtain plasma, blood samples were fractionated by centrifugation at 1,500 xg for 10 min at room temperature. After centrifugation, the plasma supernatant was immediately aliquoted, frozen and stored at −80°C until analysis. Samples collected in University of Concepción (Concepción, Chile) were shipped on dry ice to the IRBLleida Biobank.

### Antigen detection in plasma

Panbio® COVID-19 Ag Rapid Test Device (Abbott, Chicago, IL, USA) was used to detect the N antigen of SARS-CoV-2 in the plasma samples.

### MicroRNA quantification

RNA isolation and miRNA quantification were conducted in the same laboratory using standardized protocols. Experienced staff blinded to patient data performed the miRNA quantification.

Total RNA was isolated from 200 μL of frozen plasma using the miRNeasy Serum/Plasma Advanced kit (Qiagen, Hilden, Germany) according to the manufacturer’s instructions. For normalization, synthetic *Caenorhabditis elegans* miR-39-3p (cel-miR-39-3p), lacking sequence homology to human miRNAs, was added as an external reference miRNA (1.6 × 10^8^ copies/μL). The mixture was supplemented with 1 µg of MS2 carrier RNA (Roche Diagnostics, Mannheim, Germany), not containing miRNAs, to improve extracellular miRNA yield. The RNA Spike-In Kit (UniSp2, UniSp4 and UniSp5) (Qiagen, Hilden, Germany) was added to monitor RNA isolation. All reagents were spiked into samples during RNA isolation after incubation with the denaturing solution. RNA was eluted with 20 μL of RNAse-free water and stored at −80°C.

miRNA quantification was performed according to the protocol from the miRCURY LNA Universal RT microRNA PCR System (Qiagen, Hilden, Germany), which offers optimal analytical performance [26]. Reverse transcription (RT) cDNA synthesis was performed using a miRCURY LNA RT Kit (Qiagen, Hilden, Germany) in a total reaction volume of 10 μL. An additional spike-in UniSp6 (Qiagen, Hilden, Germany) was added to monitor the RT reaction. The RT reactions were performed under the following conditions: incubation for 60 min at 42°C, inactivation for 5 min at 95°C, and immediate cooling to 4°C. Then, cDNA was stored at 20°C. A panel of 41 miRNAs **(Supplemental Table S1)**, previously described [27], was analyzed using miRCURY LNA miRNA Custom Panels (384-well plates) (Qiagen, Hilden, Germany). qPCR was carried out using the QuantStudio™ 7 Flex Real-Time PCR System (Applied Biosystems, Waltham, MA, USA) in a total volume of 10 μL. RT-qPCR conditions were 95°C for 2 min, followed by 40 cycles of 95°C for 10 s and 56°C for 1 min, followed by melting curve analysis. Synthetic UniSp3 was analyzed as an interplate calibrator and qPCR control. Amplification curves were evaluated using QuantStudio Software v1.3 (Thermo Fisher Scientific, Massachusetts, USA).

The quantification cycle (Cq) was defined as the fractional cycle number at which the fluorescence exceeded a given threshold. The specificity of the qPCR was corroborated by melting curve analysis. To ensure the optimal quality of the data, the spike-in RNA templates were first analyzed to monitor the uniformity of the RNA extraction procedure and the efficiency of the RT and PCR. Hemolysis contamination was excluded as previously described [28]. miRNAs were considered to be expressed at Cq values < 35. Cqs above 35 cycles were considered undetectable and were censored at the minimum level observed for each miRNA. Relative quantification was performed using the 2^-ΔCq^ method (ΔCq = Cq_miRNA_-Cq_cel-miR-39-3p_). Expression levels were log-transformed for statistical purposes.

### Molecular pathway and Gene Ontology analyses

Bioinformatic prediction analysis was performed using the web-based computational tool DIANA-miRPath v3.0 [29]. DIANA-miRPath v3.0 combines information on manually curated experimentally validated miRNA:gene interactions from TaRBase v7.0 with the Kyoto Encyclopedia of Genes and Genomes (KEGG) database and Gene Ontology (GO) annotations (for biological processes). Enrichment analysis was performed using Fisher’s exact test (hypergeometric distribution). The false discovery rate (FDR)-adjusted *p*-value was set at <0.05. A Venn diagram was generated to represent shared and unique molecular pathways and biological processes for pulmonary function and radiologic features.

### Statistical analysis

Descriptive statistics were used to summarize the characteristics of the study population. Data are presented as the median [P_25_;P_75_] for continuous variables and as frequencies (percentage) for categorical variables. Due to the high burden of diffusion impairment and the lack of clinical cutoff for radiological features, both D_LCO_ and TSS were analyzed as tertiles and/or continuous variables. To identify a dose–response relationship between individual miRNAs and the outcomes, differential expression was based on p for trend using linear models for arrays [30]. The models included miRNA levels as outcome and study variables categorized by tertiles (transformed as: first tertile = 1; second tertile = 2; third tertile = 3). Differential expression between study groups is displayed in volcano plots. The multivariable analysis was performed using random forest (RF). D_LCO_ and TSS were considered as continuous variables. The stepwise feature selection process for the random forest (RF) [31] was based on three steps [31,32]. First, elimination of variables with low importance by ranking the average of the variable importance measure on 50 runs of RF. Second, calculation of the Out-of-bag (OOB) error rates of 50 RF runs for each nested model (from the most important variable to the model with all variables previously selected). The variables included in the model with the lowest OOB error were selected. Third, selection of the final model by performing an ascending sequence of RF that tests the inclusion of each variable selected in the second step. The relationship between the selected miRNAs and the outcomes was evaluated using generalized additive models (GAMs) with penalized cubic regression splines. The same models were used to adjust miRNA levels for age, sex, previous pulmonary disease, smoking history and the use of corticoids after hospital discharge. A sparse inverse covariance matrix using a Lasso (L1) penalty was estimated to study the correlation between miRNAs selected [33]. Correlations between continuous variables were estimated using Spearman rank correlation. Point-biserial correlations were used to calculate the correlations between dichotomous and continuous variables. The *p*-value threshold defining statistical differential expression was set at <0.05. All statistical analyses were performed using R software, version 4.0.2.

## Results

### Clinical characteristics of the patients

The study flowchart is presented in **Supplemental Figure S1**. Finally, 157 patients with ARDS secondary to SARS-CoV-2 infection and 33 patients who did not develop ARDS were available for miRNA quantification. Three samples were discarded due to insufficient plasma volume, the presence of hemolysis or low quality **(Supplemental Figure S2A & S2B)**. Five miRNAs, miR-34b-5p, miR-34c-5p, miR-124-3p, miR-208a-3p and miR-208b-3p, were not considered in the subsequent analysis due to undetectable levels in more than 80% of the samples.

Table 1 displays the characteristics of the study population. The median [P_25_;P_75_] age was 61 [53.2;66.8], and females represented 30.5% of the population. D_LCO_ was abnormal (<80% predicted) in 81.8% of the population. The TSS score showed a median [P_25_;P_75_] of 5 [2;8]. The characteristics of the non-ARDS control groups are shown in **Supplemental Table S2**.

**Table 1.** Characteristics of the study population.

Sociodemographic characteristics		
N= 154		
Age (years)	61.0 [53.2;66.8]	154
Sex		154
Female	47 (30.5%)	
BMI (kg/m^2^)	29.0 [26.1;33.3]	152
Smoking history	151	
Former	79 (52.3%)	
Non-smoker	65 (43.0%)	
Current	7 (4.64%)	
***Clinical characteristics***		
Hypertension	76 (50.0%)	152
Type II Diabetes Mellitus	30 (19.7%)	152
Obesity	61 (40.1%)	152
Cardiovascular disease	11 (7.24%)	152
Previous Chronic Lung Disease	14 (9.21%)	152
Asthma	12 (7.89%)	152
Chronic kidney disease	3 (1.97%)	152
Chronic liver disease	7 (4.61%)	152
***Baseline characteristics in hospital admission***		
Oxygen saturation (%)	92.0 [89.0;94.0]	141
PaO_2_/FiO_2_	233 [155;286]	142
SaO_2_/FiO_2_	345 [180;438]	140
***Hospital stay***		
Worst PaO_2_/FiO_2_	134.0 [91.0;186.3]	154
ARDS classification		154
Mild (201-300 mmHg)	33 (21.4%)	
Moderate (101-200 mmHg)	70 (45.5%)	
Severe (≤100 mmHg)	51 (33.1%)	
Hospital stay (days)	18.0 [11.0;31.2]	152
ICU admission	127 (82.5%)	154
ICU stay (days)	11.0 [5.00;25.0]	125
High-flow nasal cannula	94 (61.0%)	154
Invasive mechanical ventilation (IMV)	64 (42.4%)	151
IMV duration (days)	17.0 [10.0;25.5]	63
Non-IMV	85 (56.7%)	150
Non-IMV duration (days)	3.00 [2.00;5.00]	84
Prone positioning	60 (40.0%)	150
Prone positioning duration (hours)	39.0 [23.0;72.0]	56
Antibiotics	122 (81.3%)	150
Hydroxychloroquine	68 (45.0%)	151
Tocilizumab	82 (53.9%)	152
Corticoids	129 (85.4%)	151
Remdesivir	29 (19.2%)	151
Interferon beta	20 (16.8%)	119
Lopinavir/ritonavir	65 (43.0%)	151
Corticoids at hospital discharge	36 (26.1%)	138
***Post-COVID parameters***		
D_LCO_	66.2 [56.4;76.1]	154
<60	49 (31.8%)	
<80	77 (50.0%)	
≥80	28 (18.2%)	
TSS	5.00 [2.00;8.00]	151

Continuous variables are expressed as median [P_25_;P_75_]. Categorical variables are expressed as n (%). ARDS: acute respiratory distress syndrome. BMI: body mass index. D_LCO_: carbon monoxide diffusing capacity. FiO_2_: fraction of inspired oxygen. ICU: intensive care unit. IMV: invasive mechanical ventilation. PaO_2_: oxygen partial pressure. SaO_2_: arterial oxygen saturation. TSS: total severity score.

### MicroRNA profiling of the pulmonary function

The characteristics of the study population according to D_LCO_ tertiles are detailed in Table 2. Patients with low D_LCO_ levels were older and had longer hospital and ICU stays, higher requirements for prone positioning and a higher lung affectation in the chest CT **(**Table 2 **& Supplemental Figure S3)**.

**Table 2.** Characteristics of patients who entered in the inclusion criteria based on D_LCO_ tertiles.

	T1 [24.4, 60.1]	T2 (60.1, 72.3]	T3 (72.3, 100]	p-value	N
	N=51	N=50	N=53		
***Sociodemographic characteristics***					
Age (years)	64.0 [58.5;69.5]	56.5 [48.0;64.5]	59.0 [54.0;64.0]	0.026	154
Sex				0.175	154
Female	11 (21.6%)	18 (36.0%)	18 (34.0%)		
BMI (kg/m^2^)	28.7 [26.2;32.4]	29.2 [25.5;34.2]	29.3 [26.5;33.2]	0.497	152
Smoking history				0.678	151
Former	29 (56.9%)	23 (47.9%)	27 (51.9%)		
Non-smoker	20 (39.2%)	22 (45.8%)	23 (44.2%)		
Current	2 (3.92%)	3 (6.25%)	2 (3.85%)		
***Clinical characteristics***					
Hypertension	29 (56.9%)	23 (46.9%)	24 (46.2%)	0.280	152
Type II Diabetes Mellitus	10 (19.6%)	9 (18.4%)	11 (21.2%)	0.843	152
Obesity	20 (39.2%)	22 (44.9%)	19 (36.5%)	0.779	152
Cardiovascular disease	7 (13.7%)	2 (4.08%)	2 (3.85%)	0.055	152
Chronic lung disease	6 (11.8%)	5 (10.2%)	3 (5.77%)	0.294	152
Asthma	2 (3.92%)	6 (12.2%)	4 (7.69%)	0.484	152
Chronic kidney disease	1 (1.96%)	0 (0.00%)	2 (3.85%)	0.489	152
Chronic liver disease	2 (3.92%)	2 (4.08%)	3 (5.77%)	0.655	152
***Baseline characteristics in hospital admission***					
Oxygen saturation (%)	91.5 [89.0;93.0]	93.0 [90.0;95.0]	91.0 [89.0;93.0]	0.998	141
PaO_2_/FiO_2_	236 [163;295]	238 [173;305]	227 [134;276]	0.300	142
SaO_2_/FiO_2_	413 [244;438]	358 [171;438]	275 [178;429]	0.093	140
***Hospital stay***					
Worst PaO_2_/FiO_2_	122.0 [90.5;188.0]	134.0 [95.5;175.0]	134.0 [80.0;188.0]	0.984	154
ARDS classification				0.918	154
Mild (201-300 mmHg)	11 (21.6%)	9 (18.0%)	13 (24.5%)		
Moderate (101-200 mmHg)	24 (47.1%)	25 (50.0%)	21 (39.6%)		
Severe (≤100 mmHg)	16 (31.4%)	16 (32.0%)	19 (35.8%)		
Hospital stay (days)	27.0 [15.5;44.5]	15.0 [10.0;31.0]	15.0 [10.0;23.8]	0.001	152
ICU admission	41 (80.4%)	43 (86.0%)	43 (81.1%)	0.928	154
ICU stay (days)	16.0 [7.25;33.8]	9.00 [5.00;21.0]	7.50 [5.00;18.2]	0.014	125
High flow nasal cannula	31 (60.8%)	32 (64.0%)	31 (58.5%)	0.261	154
IMV	27 (52.9%)	17 (35.4%)	20 (38.5%)	0.140	151
IMV duration (days)	18.0 [10.0;31.0]	18.0 [11.8;32.2]	13.5 [9.75;18.2]	0.077	63
Non-IMV	33 (64.7%)	22 (46.8%)	30 (57.7%)	0.480	150
Non-IMV duration (days)	3.00 [2.00;6.00]	3.00 [2.00;4.00]	2.50 [1.25;3.00]	0.037	84
Prone positioning	27 (52.9%)	17 (35.4%)	16 (31.4%)	0.027	150
Prone positioning duration (hours)	34.5 [15.8;57.8]	41.5 [21.8;85.5]	37.5 [26.8;62.8]	0.451	56
Antibiotics	39 (76.5%)	43 (89.6%)	40 (78.4%)	0.800	150
Hydroxychloroquine	22 (43.1%)	24 (49.0%)	22 (43.1%)	1.000	151
Tocilizumab	30 (58.8%)	27 (55.1%)	25 (48.1%)	0.275	152
Corticoids	44 (86.3%)	43 (89.6%)	42 (80.8%)	0.427	151
Remdesivir	10 (19.6%)	9 (18.4%)	10 (19.6%)	1.000	151
Interferon beta	7 (17.9%)	7 (17.9%)	6 (14.6%)	0.691	119
Lopinavir/ritonavir	22 (43.1%)	21 (42.9%)	22 (43.1%)	1.000	151
Corticoids at hospital discharge	14 (30.4%)	11 (23.4%)	11 (24.4%)	0.708	138
***Post-COVID parameters***					
D_LCO_	51.5 [46.0;56.3]	66.0 [63.4;70.2]	80.4 [75.4;86.7]	<0.001	154
D_LCO_				<0.001	154
<60	49 (96.1%)	0 (0.00%)	0 (0.00%)		
<80	2 (3.92%)	50 (100%)	25 (47.2%)		
≥80	0 (0.00%)	0 (0.00%)	28 (52.8%)		
TSS score	9.00 [5.00;12.0]	4.50 [2.00;7.00]	3.50 [1.00;6.00]	<0.001	151

Continuous variables are expressed as median [P_25_;P_75_]. Categorical variables are expressed as n (%). ARDS: acute respiratory distress syndrome. BMI: body mass index. D_LCO_: carbon monoxide diffusing capacity. FiO_2_: fraction of inspired oxygen. ICU: intensive care unit. IMV: invasive mechanical ventilation. PaO_2_: oxygen partial pressure. SaO_2_: arterial oxygen saturation. TSS: total severity score.

**Figure 1.**
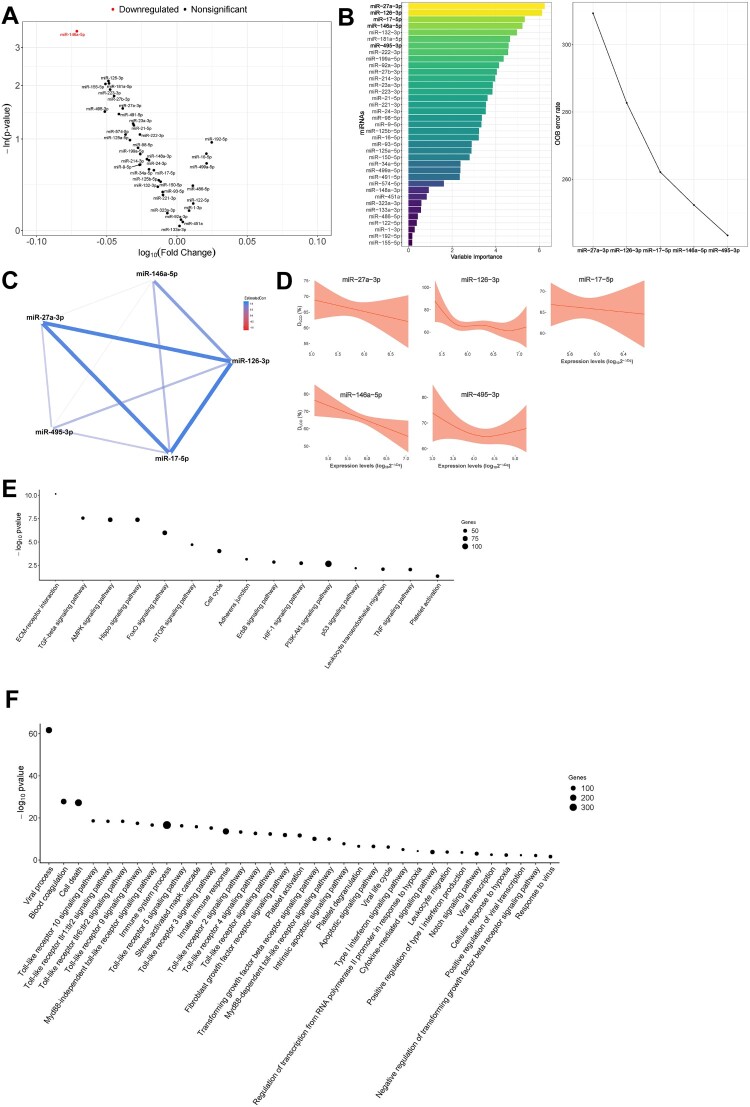
Molecular mechanisms associated with pulmonary function in survivors of SARS-CoV-2-induced ARDS. A) Volcano plot representing the *p*-value versus the fold change for each microRNA after comparison of D_LCO_ tertiles. The red dot indicates significant differences. B) Prediction model based on Random Forest. On the left, the importance of the contribution of each microRNA to the model. On the right, the best combination of microRNAs selected by the algorithm to reduce the error. C) Correlation between microRNAs that composed the signature. D) Linear or nonlinear relationship between the levels of each microRNA that composed the signature and D_LCO_. The expression levels are expressed as log_10_ (2^−ΔCq^) for statistical purposes. E) Kyoto Encyclopedia of Genes and Genomes (KEGG) analysis (selected). F) Gene Ontology (GO) analysis (selected). The *p*-value denotes the significance of the molecular pathway or the biological process and the size of the points represents the number of genes involved. The false discovery rate adjusted *p*-value cutoff was 0.05.

In univariate analyses, pulmonary function (as D_LCO_ tertiles) was inversely associated with levels of miR-146a-5p [fold change (FC) = 0.849] (Figure 1A). RF selected a miRNA profile associated with D_LCO_ levels (as continuous variable) composed of five miRNAs: miR-17-5p, miR-27a-3p, miR-126-3p, miR-146a-5p and miR-495-3p **(**Figure 1B**)**. miRNAs established a dense correlation network **(**Figure 1C**)**. The individual relation between D_LCO_ levels and the plasma miRNAs selected was analyzed using GAM modelling. miR-17-5p, miR-27a-3p and miR-146a-5p were inversely and lineally associated with D_LCO_ **(**Figure 1D**)**; high levels of these three miRNAs correlated with low to very low levels of D_LCO_, i.e. diffusion impairment. Similar results were observed after adjustment for confounders: age, sex, previous pulmonary disease, smoking history and the use of corticoids after hospital discharge **(Supplemental Figure S4A)**. These associations were not observed in non-ARDS patients positive for SARS-CoV-2 **(Supplemental Figure S5A)**. No correlation was observed between miRNA levels and invasive mechanical ventilation (IMV) duration or hospital and ICU stays **(Supplemental Figure S6)**.

Pathway and GO term enrichment analyses were performed to further understand the biological implications of the miRNA profiles described. Sixty-one KEGG pathways and one hundred and fifty-one GO terms were enriched in experimentally validated target genes of the miRNAs that composed the signature **(Supplemental Tables S3 and S4)**. The analyses identified molecular pathways and biological processes related to fibrosis (e.g. TGF-beta signalling pathway, mTOR signalling pathway, Fibroblast growth factor receptor signalling pathway), inflammation and immune response (e.g. Leukocyte transendothelial migration, TNF signalling pathway, several Toll-like receptor signalling pathways, Type I interferon signalling pathway, Cytokine-mediated signalling pathway, Leukocyte migration), cell death (e.g. Apoptotic signalling pathway), hypoxia (e.g. HIF-1 signalling pathway, Cellular response to hypoxia), vascularization (e.g. Notch signalling pathway) and coagulation (e.g. Platelet activation, Blood coagulation, Platelet degranulation) **(**Figure 1E & F**)**. Several molecular GO terms linked to viral infections were also identified (e.g. Viral process, Viral life cycle, Viral transcription, Positive regulation of viral transcription, Response to virus) **(**Figure 1E & F**)**.

### MicroRNA profiling of the radiologic features

A similar workflow was used to analyze the relation between the radiologic features and the circulating miRNA profile. Table 3 shows the characteristics of the study population according to TSS tertiles. Higher levels of TSS were directly associated with age, monocyte count, creatinine and urea levels at hospital admission, hospital stay, ICU stay, the use of IMV, IMV duration, use of prone positioning, and prone positioning duration **(**Table 3 **& Supplemental Figure S7)**.

**Table 3.** Characteristics of patients who entered in the inclusion criteria based on TSS tertiles.

	T1 [0,3]	T2 (3,7]	T3 (7,20]	p-value	N
	N=56	N=53	N=42		
***Sociodemographic characteristics***					
Age (years)	56.0 [48.0;62.0]	62.0 [56.0;67.0]	66.0 [60.2;71.0]	<0.001	151
Sex				0.285	151
Male	37 (66.1%)	37 (69.8%)	32 (76.2%)		
Female	19 (33.9%)	16 (30.2%)	10 (23.8%)		
BMI (kg/m^2^)	29.2 [26.5;34.7]	29.1 [25.6;32.9]	28.7 [27.0;32.7]	0.583	149
Smoking history				0.109	148
Former	28 (50.9%)	24 (47.1%)	26 (61.9%)		
Non-smoker	21 (38.2%)	27 (52.9%)	15 (35.7%)		
Current	6 (10.9%)	0 (0.00%)	1 (2.38%)		
***Clinical characteristics***					
Hypertension	21 (38.2%)	32 (61.5%)	22 (52.4%)	0.125	149
Type II Diabetes Mellitus	8 (14.5%)	13 (25.0%)	9 (21.4%)	0.358	149
Obesity	23 (41.8%)	22 (42.3%)	15 (35.7%)	0.566	149
Cardiovascular disease	3 (5.45%)	3 (5.77%)	5 (11.9%)	0.249	149
Chronic lung disease	5 (9.09%)	5 (9.62%)	4 (9.52%)	0.938	149
Asthma	7 (12.7%)	3 (5.77%)	2 (4.76%)	0.139	149
Chronic kidney disease	1 (1.82%)	0 (0.00%)	2 (4.76%)	0.361	149
Chronic liver disease	3 (5.45%)	1 (1.92%)	3 (7.14%)	0.769	149
***Baseline characteristics in hospital admission***					
Oxygen saturation (%)	92.0 [89.2;93.8]	92.0 [89.0;94.0]	91.5 [89.0;94.0]	0.675	139
PaO_2_/FiO_2_	220 [134;263]	248 [179;294]	229 [134;300]	0.413	139
SaO_2_/FiO_2_	325 [174;433]	410 [234;443]	332 [172;432]	0.482	138
***Hospital stay***					
Worst PaO_2_/FiO_2_	126.0 [87.5;175.0]	140.0 [118.0;176.0]	113.0 [85.2;206.0]	0.664	151
ARDS classification				0.463	151
Mild (201-300 mmHg)	11 (19.6%)	10 (18.9%)	12 (28.6%)		
Moderate (101-200 mmHg)	22 (39.3%)	32 (60.4%)	13 (31.0%)		
Severe (≤100 mmHg)	23 (41.1%)	11 (20.8%)	17 (40.5%)		
Hospital stay (days)	15.0 [10.0;24.5]	17.5 [10.8;31.2]	26.5 [17.0;45.8]	0.001	149
ICU admission	46 (82.1%)	43 (81.1%)	37 (88.1%)	0.465	151
ICU stay (days)	6.00 [3.00;12.8]	6.00 [3.75;17.5]	15.0 [5.50;32.5]	0.003	148
High flow nasal cannula	31 (55.3%)	38 (71.7%)	24 (57.2%)	0.849	151
IMV	17 (30.9%)	23 (45.1%)	24 (57.1%)	0.010	148
IMV duration (days)	0.00 [0.00;5.00]	0.00 [0.00;13.0]	8.00 [0.00;25.0]	0.002	147
Non-IMV	29 (53.7%)	27 (52.9%)	28 (66.7%)	0.226	147
Non-IMV duration (days)	1.00 [0.00;3.00]	1.00 [0.00;3.00]	2.50 [0.00;4.75]	0.061	146
Prone positioning	16 (29.6%)	19 (37.3%)	25 (59.5%)	0.004	147
Prone positioning duration (hours)	0.00 [0.00;5.00]	0.00 [0.00;19.5]	17.5 [0.00;47.5]	<0.001	143
Antibiotics	46 (85.2%)	40 (78.4%)	36 (85.7%)	0.991	147
Hydroxychloroquine	31 (57.4%)	19 (36.5%)	18 (42.9%)	0.125	148
Tocilizumab	26 (47.3%)	31 (59.6%)	23 (54.8%)	0.417	149
Corticoids	44 (80.0%)	46 (90.2%)	36 (85.7%)	0.380	148
Remdesivir	7 (13.0%)	10 (19.2%)	10 (23.8%)	0.170	148
Interferon beta	5 (12.5%)	6 (14.6%)	9 (24.3%)	0.172	118
Lopinavir/ritonavir	28 (51.9%)	19 (36.5%)	18 (42.9%)	0.330	148
Corticoids at discharge	9 (18.8%)	16 (32.7%)	10 (26.3%)	0.295	135
***Post-COVID parameters***					
D_LCO_	71.3 [65.0;81.6]	70.0 [56.6;78.1]	54.7 [48.1;62.5]	<0.001	151
D_LCO_				<0.001	151
<60	7 (12.5%)	14 (26.4%)	26 (61.9%)		
<80	32 (57.1%)	29 (54.7%)	15 (35.7%)		
≥80	17 (30.4%)	10 (18.9%)	1 (2.38%)		
TSS score	2.00 [0.00;2.25]	5.00 [5.00;7.00]	11.0 [10.0;13.0]	<0.001	151

Continuous variables are expressed as median [P_25_;P_75_]. Categorical variables are expressed as n (%). ARDS: acute respiratory distress syndrome. BMI: body mass index. D_LCO_: carbon monoxide diffusing capacity. FiO_2_: fraction of inspired oxygen. ICU: intensive care unit. IMV: invasive mechanical ventilation. PaO_2_: oxygen partial pressure. SaO_2_: arterial oxygen saturation. TSS: total severity score.

**Figure 2.**
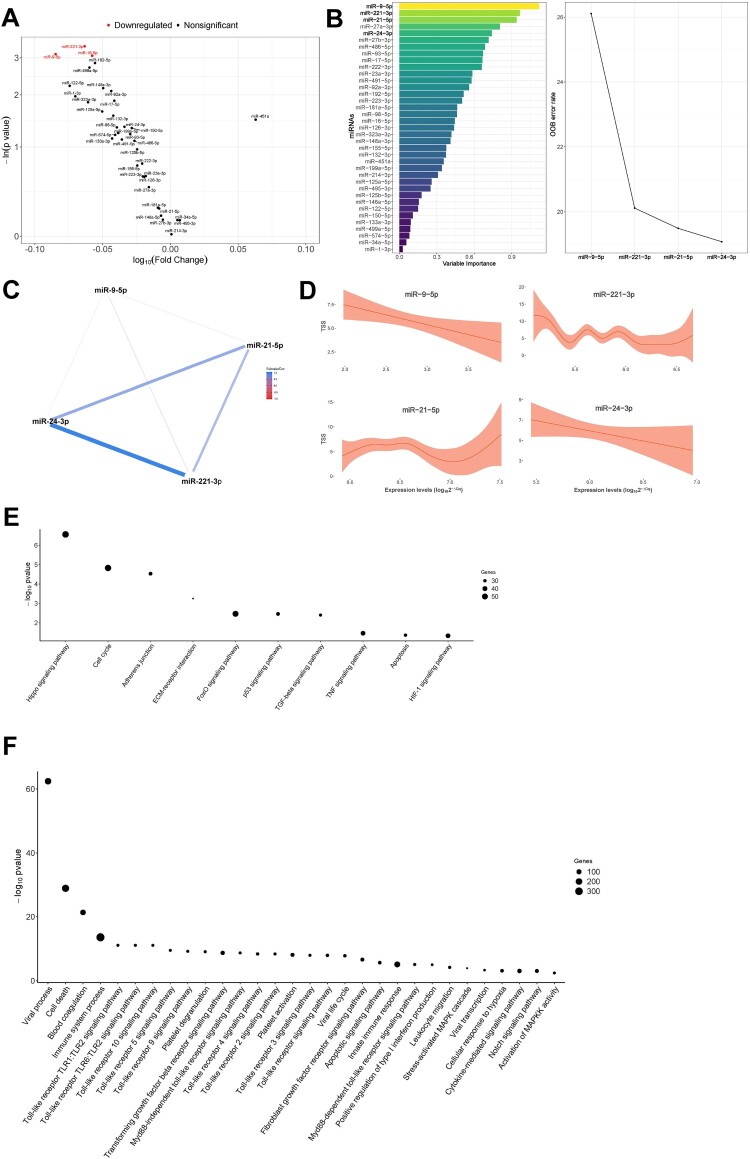
Molecular mechanisms associated with radiologic features in survivors of SARS-CoV-2-induced ARDS. A) Volcano plot representing the *p*-value versus the fold change for each microRNA after comparison of TSS tertiles. The red dots indicate significant differences. B) Prediction model based on Random Forest. On the left, the importance of the contribution of each microRNA to the model. On the right, the best combination of microRNAs selected by the algorithm to reduce the error. C) Correlation between microRNAs that composed the signature. D) Linear or nonlinear relationship between the levels of each microRNA that composed the signature and D_LCO_. The expression levels are expressed as log_10_ (2^−ΔCq^) for statistical purposes. E) Kyoto Encyclopedia of Genes and Genomes (KEGG) analysis (selected). F) Gene Ontology (GO) analysis (selected). The *p*-value denotes the significance of the molecular pathway or the biological process and the size of the points represents the number of genes implicated. The false discovery rate adjusted *p*-value cutoff was 0.05.

In univariate analyses, miR-9-5p [FC = 0.823], miR-16-5p [FC = 0.876] and miR-221-3p [FC = 0.864] were inversely associated with TSS (as tertiles) (Figure 2A). Following the same approach, the feature selection procedure constructed a signature composed of four miRNAs: miR-9-5p, miR-21-5p, miR-24-3p and miR-221-3p (using TSS as continuous variable) **(**Figure 2B**)**. Except for miR-9-5p, which was poorly correlated with miR-221-3p, all miRNAs correlated with each other **(**Figure 2C**)**. GAM modelling showed that low levels of both miR-9-5p and miR-24-3p adjusted to an inverse linear relation with TSS and, consequently, higher levels of radiologic abnormalities. A nonlinear relationship was observed for miR-21-5p and miR-221-3p **(**Figure 2D**)**. Confounding factors showed no significant impact on the miRNA-TSS relationship **(Supplemental Figure S4B)**. Except for miR-24-3p, the relationship between TSS levels and the selected miRNAs was only observed in the ARDS group **(Supplemental Figure S5B)**. Again, no correlation was observed between IMV duration or hospital and ICU stays and miRNA levels **(Supplemental Figure S6)**.

**Figure 3.**
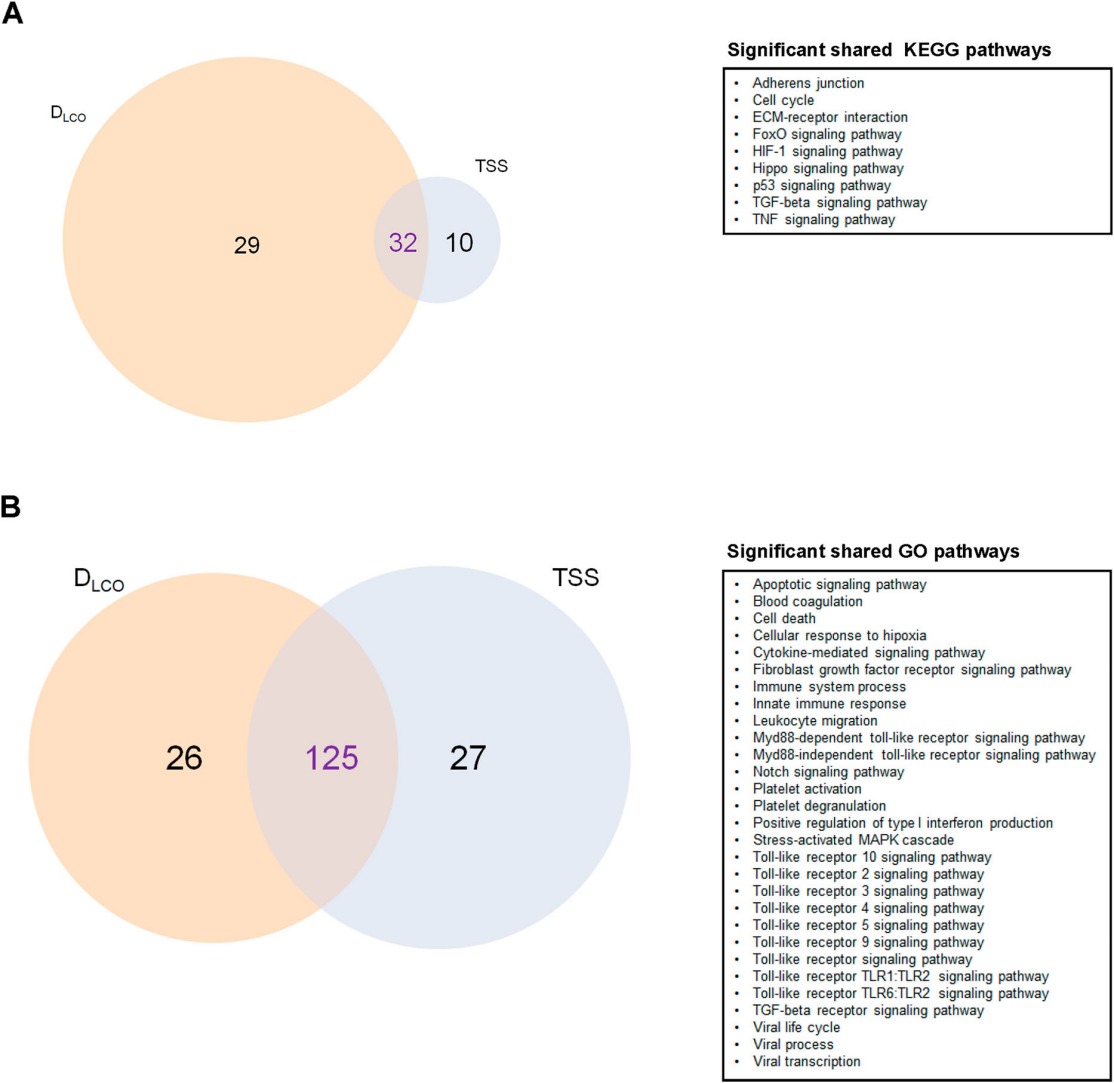
Molecular mechanisms associated with pulmonary function and/or radiologic features. A) Venn diagram displaying the shared and unique Kyoto Encyclopedia of Genes and Genomes (KEGG) pathways (selected). B) Venn diagram displaying the shared and unique Gene Ontology (GO) terms (selected). The size of each circle is proportional to the total number of molecular pathways or biological processes related to D_LCO_ and TSS. On the right, significant shared KEGG pathways and GO terms are reported for each diagram.

Forty-two KEGG pathways and 115 GO terms contained experimentally validated miRNA:gene interactions **(Supplemental Tables S5 and S6)**. Again, the analyses identified molecular pathways and biological processes related to fibrosis (e.g. TGF-beta signalling pathway, Fibroblast growth factor receptor signalling pathway), inflammation and immune response (e.g. TNF signalling pathway, several Toll-like receptor signalling pathways, Leukocyte migration), cell death (e.g. Apoptotic signalling pathway), hypoxia (e.g. HIF-1 signalling pathway, Cellular response to hypoxia), vascularization (e.g. Notch signalling pathway) and coagulation (e.g. Blood coagulation, Platelet degranulation, Platelet activation) and viral infections (e.g. Viral process, Viral life cycle, Viral transcription) **(**Figure 2E & F**)**. An overlap between those molecular pathways and GO terms enriched in targets from the miRNA signatures associated with lung function and radiologic features was observed (Figure 3).

Molecular mechanisms involved in viral infection and response were highly enriched among the identified GO terms for both signatures. Therefore, we evaluated the presence of viral antigens in plasma samples from a subset of 50 survivors of SARS-CoV-2-induced ARDS. Antigenemia was presented in two patients **(Supplemental Figure S8)**.

## Discussion

Current study combined experimental and computational approaches to first evaluate the association between lung function and radiologic features and the circulating miRNA profile in survivors of SARS-CoV-2-induced ARDS 3 months after hospital discharge and then decipher the molecular mechanisms implicated in pulmonary outcomes. We report four major findings: i) distinct miRNA profiles are associated with pulmonary function and radiologic features; ii) this association seems to be specific for ARDS secondary to SARS-CoV-2 infection; iii) pulmonary function and structural features are linked not only to mechanisms such as fibrosis or inflammation but also to cell death, immune response, hypoxia, vascularization, coagulation and viral infection; iv) as such, the mechanisms linked to the respiratory sequelae of SARS-CoV-2-induced ARDS might implicate multiple biological pathways.

Individual miRNAs have a modest impact on the regulation of biological processes [34]. Collective and coordinated miRNA expression networks, rather than single miRNAs, are critical for the regulation of the cellular phenotype [13]. The analysis of miRNA profiles is paramount. Furthermore, the deregulation in individual expression programmes may not explain the complexity of the disease. For instance, Barbagallo et al. [35] have recently demonstrated a profound perturbation in competitive endogenous RNA networks due to SARS-CoV-2 infection which ultimately impacts on disease progression. Advanced statistical tools seem fundamental to describe the interaction between miRNAs and the phenotype and identify nonlinear associations between miRNA levels and the outcome. Consequently, we constructed miRNA signatures using machine learning models. RF-based predictive models identified two miRNA signatures associated with pulmonary function and radiologic features in survivors of ARDS secondary to SARS-CoV-2 infection. The current results could improve the limited understanding of sequelae in survivors of SARS-CoV-2-induced ARDS. Supporting the significant functional redundancy among miRNAs [36], although the individual miRNAs that composed the patterns were different, the downstream regulatory effects were highly similar with miR-17-5p, miR-27a-3p, miR-126-3p, miR-146a-5p and miR-495-3p for D_LCO_ and miR-9-5p, miR-21-5p, miR-24-3p and miR-221-3p for TSS.

As expected, both pulmonary function and radiological features were closely linked to fibrotic and inflammatory pathways. The reduction in miR-9-5p, a suppressor of TGF-β1-dependent myofibroblast phenotypic transformation in lung disease [37], may indicate a profibrotic state in survivors of severe forms of COVID-19. The results in subjects with persistent symptoms after acute COVID-19 [38] and bioinformatic prediction using experimentally validated miRNA interactions corroborated this notion. Different cellular pathways related to fibrotic mechanisms in lung conditions were identified, including the AMPK signalling pathway [39], Hippo signalling pathway [40] and mTOR signalling pathway [41]. Concerning inflammation, multiple KEGG pathways and GO terms, such as signalling pathways involving TNF or Toll-like receptors, were enriched in the target genes of the miRNA patterns. More specifically, the levels of miR-146a-5p were closely and inversely associated with lung function. This miRNA is induced in response to proinflammatory cytokines and participates in a negative feedback regulation loop to control inflammation [42]. Therefore, its increase in survivors with higher pulmonary impairment may constitute an anti-inflammatory response to the extensive inflammatory insult inherent to the pathogenesis of ARDS, similar to the production of anti-inflammatory cytokines in proinflammatory conditions. In fact, hypercytokinemia is a characteristic feature of severe COVID-19 [43], and its prolongation even 40–60 days postviral infection has been reported [44]. The biological functions of the other miRNAs that composed the signatures support this hypothesis. *In vitro* and *in vivo* approaches suggest that mesenchymal stem cell-derived extracellular vesicles mitigate acute lung injury at least partially by transferring miR-27a-3p to alveolar macrophages, a regulator of NFKB1 and M2 macrophage polarization [45]. Endothelial progenitor cell exosomes reduce local inflammatory cytokines in a model of lipopolysaccharide-induced acute lung injury, in part through the delivery of miR-126 [46].

The circulating miRNA signature may be informative about other driving factors that mediate sequelae from SARS-CoV-2-induced ARDS. KEGG and GO analyses highlighted pathways related to platelet function and blood coagulation. SARS-CoV-2 infection induces a pro-coagulant state that has been recognized as a hallmark of COVID-19 [47]. We have previously demonstrated alterations in the circulating profile of miRNAs implicated in coagulation mechanisms during the acute phase of the disease [27]. According to our findings, the pathways related to the coagulation process remain perturbed even 3 months after hospital discharge. Although the clinical relevance remains uncertain [48], these results are in line with the occurrence of thrombotic events after discharge or resolution of infection [49]. In addition, miRNA signatures were enriched in targets of signalling pathways implicated in cell death/apoptosis (e.g. FoxO signalling pathway or p53 signalling pathway, among others), indicating that the cell injury mechanisms modulated during the acute phase by SARS-CoV-2 [50] are still deregulated in “post-COVID” syndrome. Interestingly, the increased level of miR-126-3p in patients with significant diffusion impairment is compatible with aberrant angiogenic signalling. Cao et al. [51] recently demonstrated that miR-126-3p inhibits the angiogenic function of human lung microvascular endothelial cells, which are essential for gas exchange and for lung injury repair and regeneration. Current results may have a relevant impact, since the mechanisms that mediate microvascular injury in ARDS sequelae are uncertain [52]. Possible evidence for the phenotypic implications of our findings is given by the identification of target genes in hypoxia signalling pathways. The perturbations in these mechanisms may explain the decreased average, final and minimal oxygen saturation in the 6-minute walking test that we have previously described in survivors of SARS-CoV-2-induced ARDS [6]. Of note, the miRNA signatures were also enriched in targets from GO terms related to viral infection. Despite this fact, we have not detected the virus in the blood through antigenemia profiling. A plausible explanation is the presence of viral fragments in other tissues causing immunoreactivity and persistent inflammation [53]. The alteration of these biological processes during the recovery phase deserves additional investigation.

Although the endocrine function of circulating miRNAs is still not completely elucidated [54], our work suggests a potential role of miRNAs in the respiratory sequelae of severe COVID-19. Overall, the current results provide a framework for targeted interventions against the sequelae of patients who develop SARS-CoV-2-induced ARDS. The management of the long-lasting effects of this syndrome should be based on a comprehensive and multidisciplinary approach to not only focus on inflammation and fibrosis but also other relevant pathological processes.

Our results should be considered in the context of several limitations. First, survivors of ARDS secondary to SARS-CoV-2 infection were recruited in a single centre. The results should be validated in independent cohorts. Second, potential confounding due to previous comorbidities, clinical management and inpatient complications should not be discarded. Third, how the deregulation of the miRNA signature in plasma impacts the pulmonary sequelae remains to be elucidated. Further functional studies may provide additional insights into role of the miRNAs in pulmonary abnormalities. Forth, we used a targeted approach using miRNAs previously associated with COVID-19 and/or pathological mechanisms linked to COVID-19/ARDS [27]. Other miRNAs could be associated with pathophysiology of the postacute pulmonary sequelae. Large-scale miRNA analyses are fundamental. Fifth, potential overfitting should be acknowledged. In addition, the impact of type I error should not be discarded.

In conclusion, the pulmonary function and radiologic features in survivors of ARDS secondary to SARS-CoV-2 infection are associated with specific plasma miRNA patterns. The multifactorial mechanisms linked to the miRNA profiles provide novel knowledge of the physiopathology of persistent pulmonary dysfunction in the recovery stage and, in consequence, further insights into postacute care strategies.

## Supplementary Material

Supplemental Material
