# Supplementary material for: Identification of circulating microRNA profiles associated with pulmonary function and radiologic features in survivors of SARS-CoV-2-induced ARDS
Source: Emerg Microbes Infect. 2022 Jun 4;11(1):1537–49. doi: 10.1080/22221751.2022.2081615 (PMC9176679; doi:10.1080/22221751.2022.2081615)
Supplement: Supplemental Material [file TEMI_A_2081615_SM1829.docx]

**Supplemental online material**

**Identification of circulating microRNA profiles associated with pulmonary function and radiologic features in survivors of SARS-CoV-2-induced ARDS**

María C. García-Hidalgo,^1^ Jessica González,^1,2^ Iván D. Benítez,^1,2^ Paola Carmona,^1^ Sally Santisteve,^1^ Manel Pérez-Pons,^1,2^ Anna Moncusí-Moix,^1,2^ Clara Gort-Paniello,^1,2^ Fátima Rodríguez-Jara,^1^ Marta Molinero,^1^ Thalia Belmonte,^1,2^ Gerard Torres,^1,2^ Gonzalo Labarca,^3,4^ Estefania Nova-Lamperti,^3^ Jesús Caballero,^5^ Jesús F. Bermejo-Martin,^2,6^ Adrián Ceccato,^2^ Laia Fernández-Barat,^2,7^ Ricard Ferrer,^2,8^ Dario Garcia-Gasulla,^9^ Rosario Menéndez,^2,10^ Ana Motos,^2,7^ Oscar Peñuelas,^2,11^ Jordi Riera,^2,8^ Antoni Torres,^2,7^ Ferran Barbé,^1,2^ David de Gonzalo-Calvo,^1,2,*^

*on behalf of the CIBERESUCICOVID Project (COV20/00110, ISCIII).*

^1^ Translational Research in Respiratory Medicine, University Hospital Arnau de Vilanova and Santa Maria, IRBLleida, Lleida, Spain.

^2^ CIBER of Respiratory Diseases (CIBERES), Institute of Health Carlos III, Madrid, Spain.

^3^ Molecular and Translational Immunology Laboratory, Department of Clinical Biochemistry and Immunology, Faculty of Pharmacy, Universidad de Concepcion, Concepcion, Chile.

^4^ Internal Medicine Unit, Complejo Asistencial Dr. Víctor Ríos Ruiz, Los Ángeles, Chile.

^5^ Intensive Care Department, University Hospital Arnau de Vilanova, IRBLleida, Lleida, Spain. ^6^ Hospital Universitario Río Hortega de Valladolid, Valladolid, Spain; Instituto de Investigación Biomédica de Salamanca (IBSAL), Salamanca, Spain.

^7^ Servei de Pneumologia, Hospital Clinic; Universitat de Barcelona; IDIBAPS, Barcelona, Spain.

^8^ Intensive Care Department, Vall d’Hebron Hospital Universitari. SODIR Research Group, Vall d’Hebron Institut de Recerca (VHIR), Spain.

^9^ Barcelona Supercomputing Center (BSC), Barcelona, Spain.

^10^ Pulmonology Service, University and Polytechnic Hospital La Fe, Valencia, Spain.

^11^ Hospital Universitario de Getafe, Madrid, Spain.

**Correspondence to:**

David de Gonzalo-Calvo, Ph.D.

Translational Research in Respiratory Medicine, University Hospital Arnau de Vilanova and Santa Maria, IRBLleida.

Avda Alcalde Rovira Roure 80 · 25198 Lleida, Spain.

E-mail: dgonzalo@irblleida.cat

**Supplemental Figures**


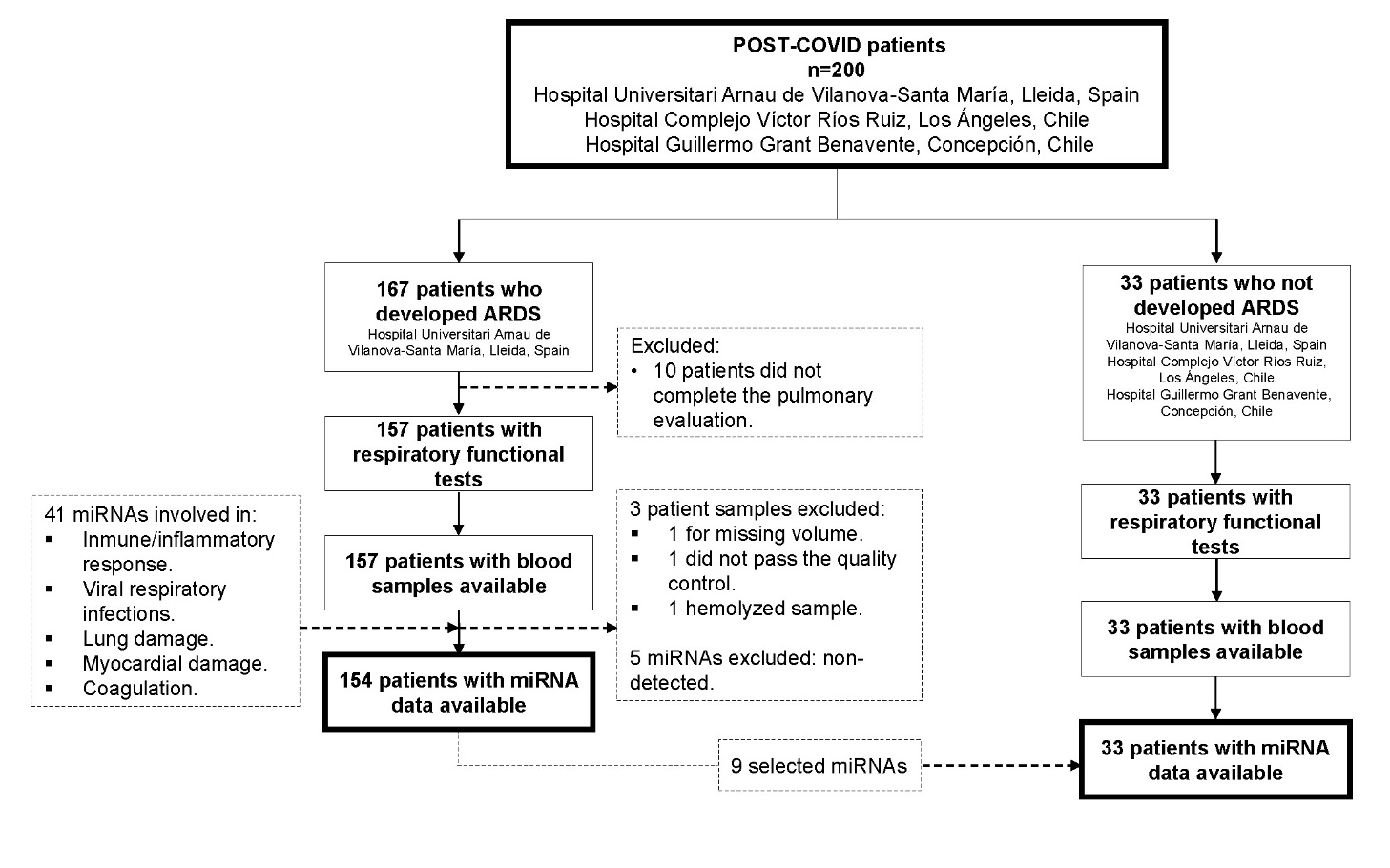


**Supplemental Figure S1.** Study flowchart. A total of 167 patients infected by SARS-CoV-2 in the first and second waves who developed ARDS as a consequence attended follow-up. After excluding patients who did not complete respiratory functional tests, 157 samples were available for microRNA quantification. Three samples were not included in the final analyses due to missing volume, hemolysis and high variability in quality control. Additionally, we did not include five nondetected microRNAs (miR-34b-5p, miR-34c-5p, miR-124-3p, miR-208a-3p and miR-208b-3p). A control cohort (n=33), composed by patients positive for SARS-CoV-2 but who did not develop ARDS was included to analyze the specificity of the associations between circulating miRNA profiles and pulmonary function and radiologic features in survivors of SARS-CoV-2-induced ARDS.


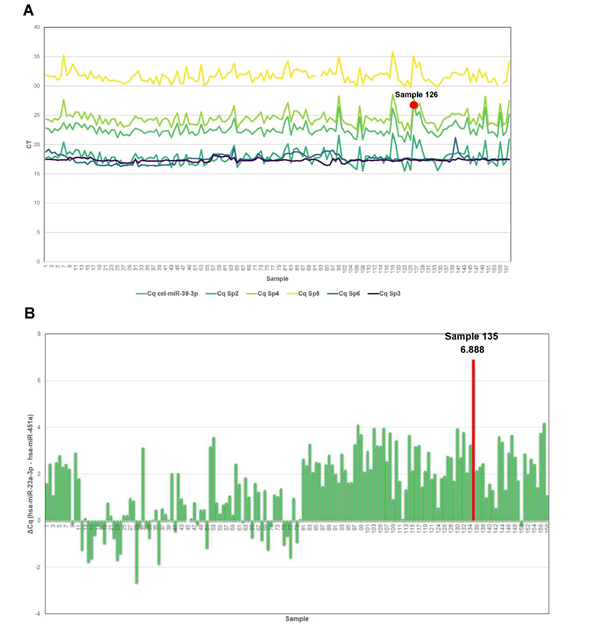
 **Supplemental Figure S2.** Quality control. A) Spike-in levels. B) Hemolysis test.


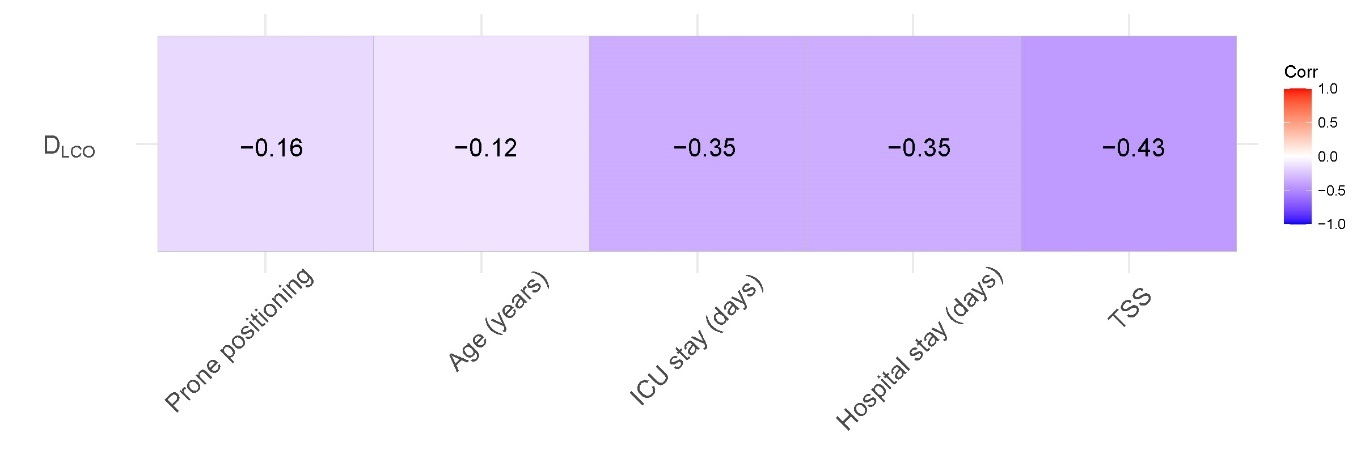


**Supplemental Figure S3.** Correlation between D_LCO_ levels and demographic and clinical variables. Point-biserial correlation was used to estimate the correlation between dichotomous and continuous variables, represented as r_pb_ values, and Spearman’s test was used to estimate the correlation between continuous variables, represented as rho values.


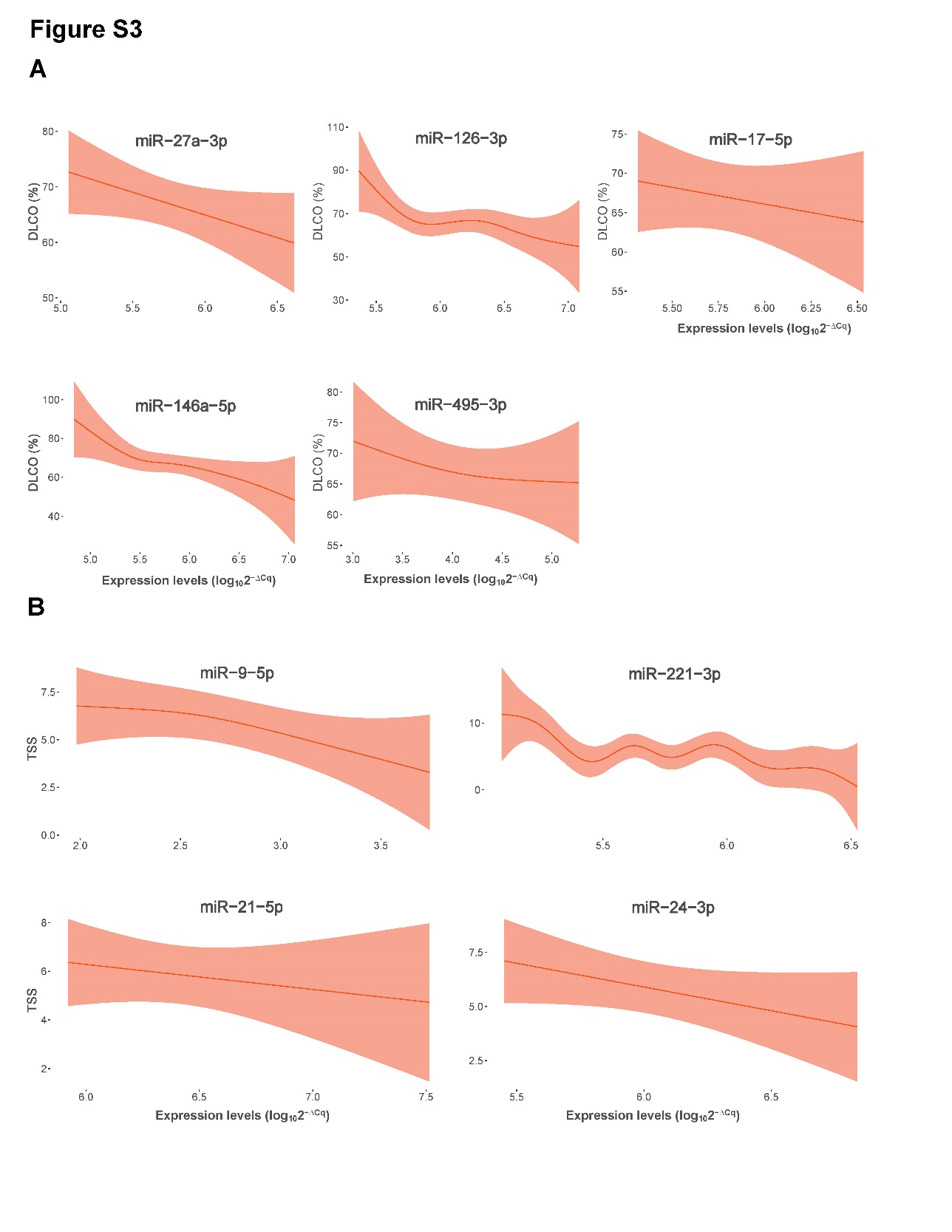


**Supplemental Figure S4.** Age, sex, previous chronic pulmonary disease, smoking history and the use of corticoids after hospital discharge showed no significant impact on the association between microRNA levels and pulmonary function or radiologic features. A) Representation of the linear or nonlinear relationship between the value of DLCO and the expression levels of each of the microRNAs of the signature adjusted by age, sex, previous chronic pulmonary disease, smoking history and the use of corticoids after hospital discharge. B) Representation of the linear or nonlinear relationship between the value of TSS and the expression levels of each of the microRNAs of the signature adjusted by age, sex, previous chronic pulmonary disease, smoking history and the use of corticoids after hospital discharge. microRNA expression levels are expressed as log_10_ (2^−∆Cq^) for statistical purposes.

**
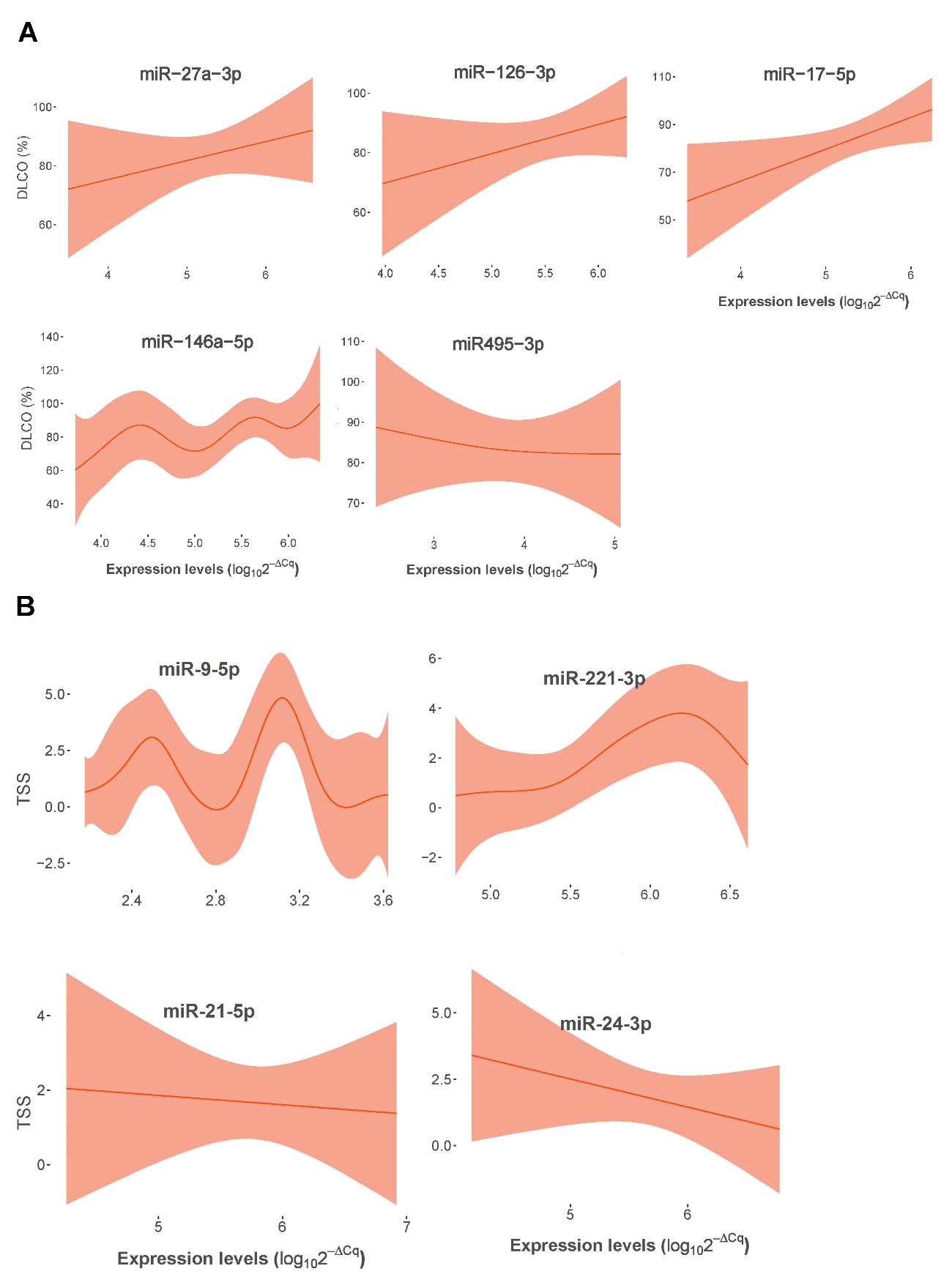
**

**Supplemental Figure S5.** Linear or nonlinear relationship between the levels of each microRNA that composed the signatures and diffusion capacity and radiological features, respectively, in patients positive for SARS-CoV-2 who did developed ARDS. A) GAM modeling for D_LCO_ (Y axis) and expression levels (expressed as log_10_ 2^−∆Cq^) (X axis). B) GAM modeling for TSS (Y axis) and expression levels (expressed as log_10_ 2^−∆Cq^) (X axis).

**
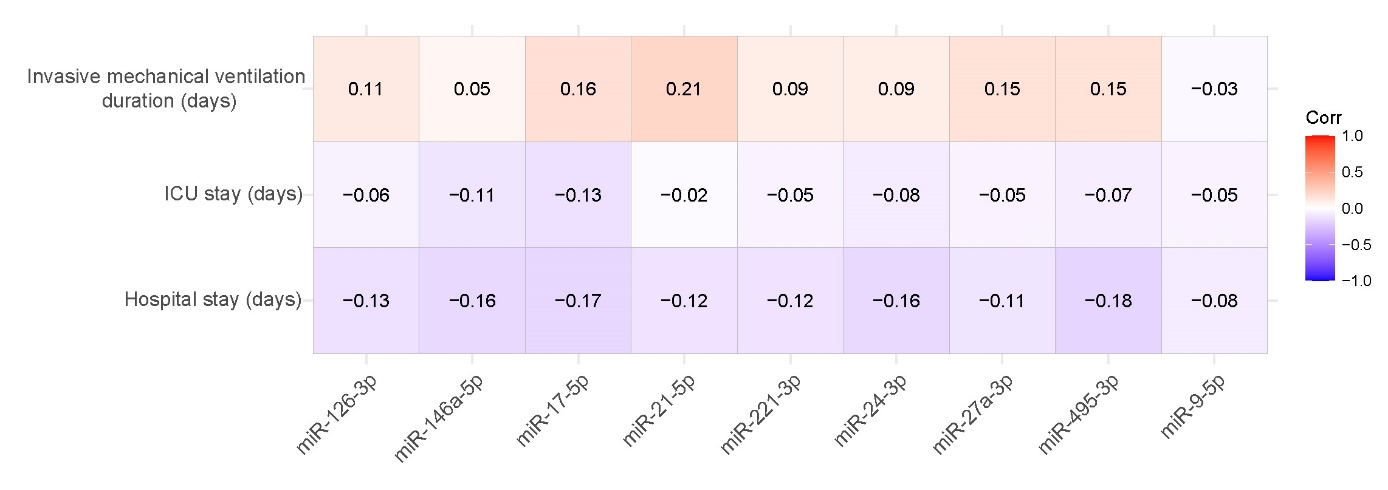
**

**Supplemental Figure S6**. Correlation between individual miRNAs in both signatures and clinical variables related to the severity of the acute phase. Spearman’s test was used to calculate the correlation between the variables, represented as rho values.


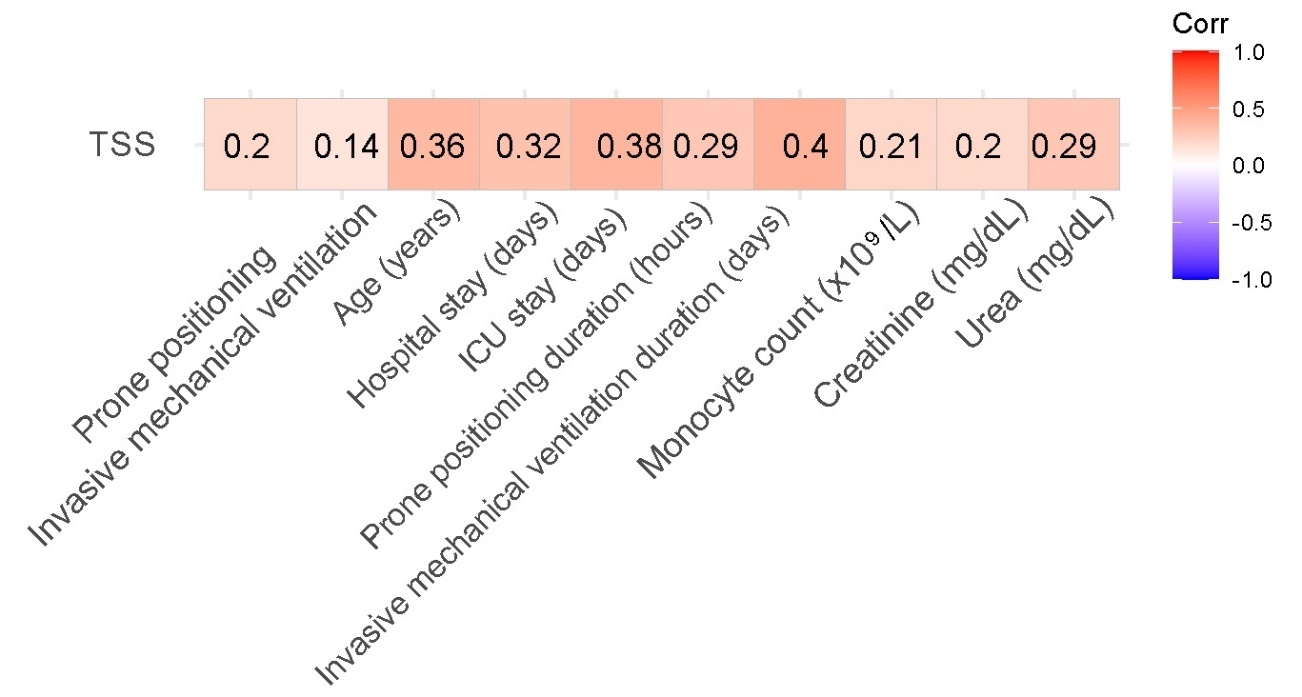


**Supplemental Figure S7.** Correlation between TSS levels and demographic and clinical variables. Point-biserial correlation was used to estimate the correlation between dichotomous and continuous variables, represented as r_pb_ values, and Spearman’s test was used to estimate the correlation between continuous variables, represented as rho values.


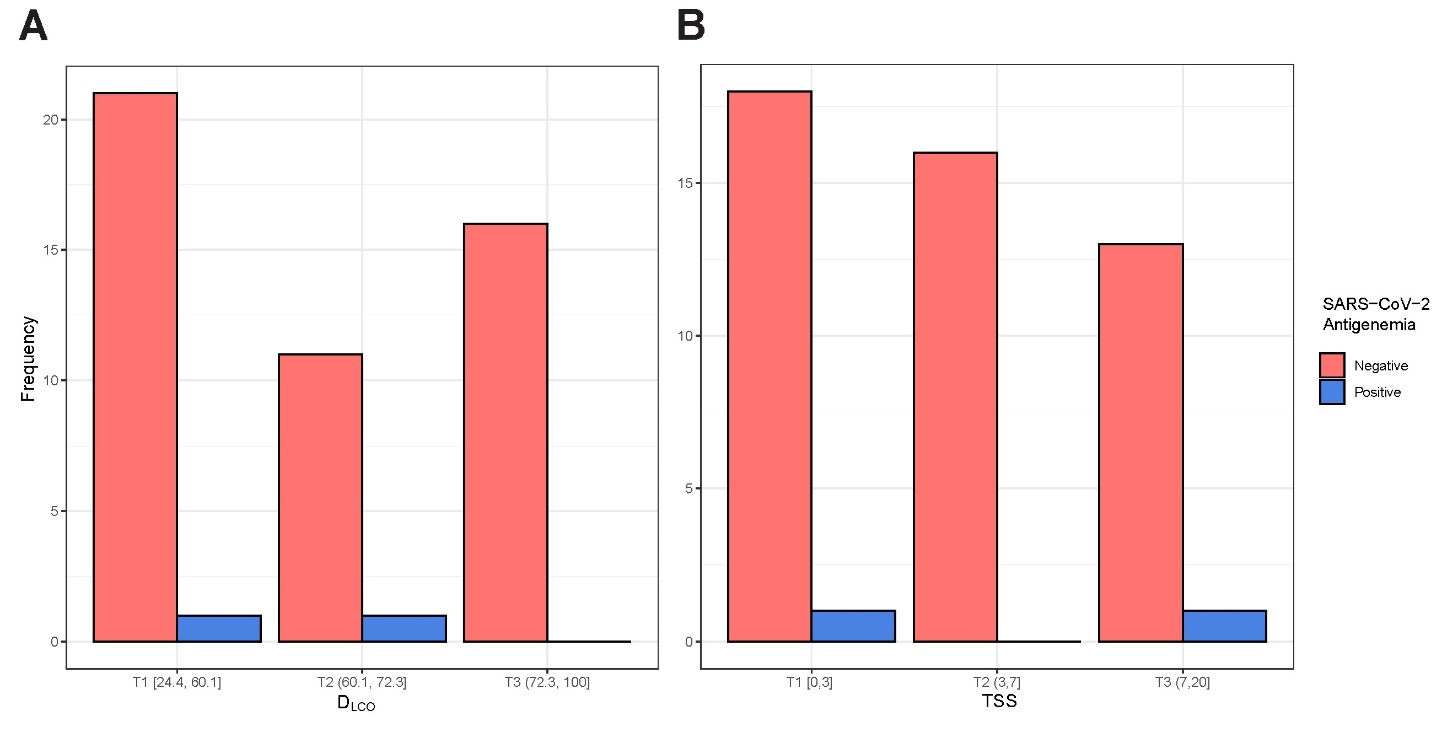


**Supplemental Figure S8.** SARS-CoV-2 antigenemia in plasma. A) SARS-CoV-2 antigen detection in plasma samples according to D_LCO_ tertiles. B) SARS-CoV-2 antigen detection in plasma samples according to TSS tertiles.

**Supplemental Tables**

**Supplemental Table S1.** Panel of miRNAs analyzed in the study population.

| **microRNA ID** | **miRBase Accession Number** | **MiRCury Assay** | **Target sequence** |
| --- | --- | --- | --- |
| hsa-miR-1-3p | MIMAT0000416 | YP00204344 | UGGAAUGUAAAGAAGUAUGUAU |
| hsa-miR-9-5p | MIMAT0000441 | YP00204513 | UCUUUGGUUAUCUAGCUGUAUGA |
| hsa-miR-16-5p | MIMAT0000069 | YP00205702 | UAGCAGCACGUAAAUAUUGGCG |
| hsa-miR-17-5p | MIMAT0000070 | YP02119304 | CAAAGUGCUUACAGUGCAGGUAG |
| hsa-miR-21-5p | MIMAT0000076 | YP00204230 | UAGCUUAUCAGACUGAUGUUGA |
| hsa-miR-24-3p | MIMAT0000080 | YP00204260 | UGGCUCAGUUCAGCAGGAACAG |
| hsa-miR-27a-3p | MIMAT0000084 | YP00206038 | UUCACAGUGGCUAAGUUCCGC |
| hsa-miR-27b-3p | MIMAT0000419 | YP00205915 | UUCACAGUGGCUAAGUUCUGC |
| hsa-miR-34a-5p | MIMAT0000255 | YP00204486 | UGGCAGUGUCUUAGCUGGUUGU |
| hsa-miR-34b-5p | MIMAT0000685 | YP00204424 | UAGGCAGUGUCAUUAGCUGAUUG |
| hsa-miR-34c-5p | MIMAT0000686 | YP00205659 | AGGCAGUGUAGUUAGCUGAUUGC |
| hsa-miR-92a-3p | MIMAT0000092 | YP00204258 | UAUUGCACUUGUCCCGGCCUGU |
| hsa-miR-93-5p | MIMAT0000093 | YP00204715 | CAAAGUGCUGUUCGUGCAGGUAG |
| hsa-miR-98-5p | MIMAT0000096 | YP00204640 | UGAGGUAGUAAGUUGUAUUGUU |
| hsa-miR-122-5p | MIMAT0000421 | YP00205664 | UGGAGUGUGACAAUGGUGUUUG |
| hsa-miR-124-3p | MIMAT0000422 | YP00206026 | UAAGGCACGCGGUGAAUGCC |
| hsa-miR-125a-5p | MIMAT0000443 | YP00204339 | UCCCUGAGACCCUUUAACCUGUGA |
| hsa-miR-125b-5p | MIMAT0000423 | YP00205713 | UCCCUGAGACCCUAACUUGUGA |
| hsa-miR-126-3p | MIMAT0000445 | YP00204227 | UCGUACCGUGAGUAAUAAUGCG |
| hsa-miR-132-3p | MIMAT0000426 | YP00206035 | UAACAGUCUACAGCCAUGGUCG |
| hsa-miR-133a-3p | MIMAT0000427 | YP00204788 | UUUGGUCCCCUUCAACCAGCUG |
| hsa-miR-146a-5p | MIMAT0000449 | YP00204688 | UGAGAACUGAAUUCCAUGGGUU |
| hsa-miR-148a-3p | MIMAT0000243 | YP00205867 | UCAGUGCACUACAGAACUUUGU |
| hsa-miR-150-5p | MIMAT0000451 | YP00204660 | UCUCCCAACCCUUGUACCAGUG |
| hsa-miR-155-5p | MIMAT0000646 | YP00204308 | UUAAUGCUAAUCGUGAUAGGGGU |
| hsa-miR-181a-5p | MIMAT0000256 | YP00206081 | AACAUUCAACGCUGUCGGUGAGU |
| hsa-miR-192-5p | MIMAT0000222 | YP00204099 | CUGACCUAUGAAUUGACAGCC |
| hsa-miR-199a-5p | MIMAT0000231 | YP00204494 | CCCAGUGUUCAGACUACCUGUUC |
| hsa-miR-208a-3p | MIMAT0026474 | YP00205619 | AUAAGACGAGCAAAAAGCUUGU |
| hsa-miR-208b-3p | MIMAT0026722 | YP00204636 | AUAAGACGAACAAAAGGUUUGU |
| hsa-miR-214-3p | MIMAT0000271 | YP00204510 | ACAGCAGGCACAGACAGGCAGU |
| hsa-miR-221-3p | MIMAT0000278 | YP00204532 | AGCUACAUUGUCUGCUGGGUUUC |
| hsa-miR-222-3p | MIMAT0000279 | YP00204551 | AGCUACAUCUGGCUACUGGGU |
| hsa-miR-223-3p | MIMAT0000280 | YP00205986 | UGUCAGUUUGUCAAAUACCCCA |
| hsa-miR-323a-3p | MIMAT0000755 | YP00204278 | CACAUUACACGGUCGACCUCU |
| hsa-miR-451a | MIMAT0001631 | YP02119305 | AAACCGUUACCAUUACUGAGUU |
| hsa-miR-486-5p | MIMAT0002177 | YP00204001 | UCCUGUACUGAGCUGCCCCGAG |
| hsa-miR-491-5p | MIMAT0002807 | YP00204695 | AGUGGGGAACCCUUCCAUGAGG |
| hsa-miR-495-3p | MIMAT0002817 | YP00206015 | AAACAAACAUGGUGCACUUCUU |
| hsa-miR-499a-5p | MIMAT0002870 | YP00205935 | UUAAGACUUGCAGUGAUGUUU |
| hsa-miR-574-5p | MIMAT0004795 | YP02116206 | UGAGUGUGUGUGUGUGAGUGUGU |

**Supplemental Table S2**. Characteristics of non-ARDS patients attended to Post-COVID evaluation.

|  | N=33 | N |
| --- | --- | --- |
| ***Sociodemographic characteristics*** | | |
| Age (years), median [P_25_;P_75_] | 48.0 [37.0;61.0] | 33 |
| Sex, n(%): |  | 33 |
| Female | 17 (51.5%) |  |
| Male | 16 (48.5%) |  |
| ***Hospital stay*** | | |
| Hospital admission | 18 (54.5%) | 33 |
| ***Post-COVID parameters*** | | |
| D_LCO_, median [P_25_;P_75_] | 86.0 [67.3;94.0] | 33 |
| <60, n (%) | 3 (9.09%) |  |
| <80, n (%) | 19 (57.6%) |  |
| >=80, n (%) | 11 (33.3%) |  |
| TSS score, median [P_25_;P_75_] | 0.00 [0.00;2.00] | 33 |

Continuous variables are expressed as median [P_25_;P_75_]. Categorical variables are expressed as n (%). D_LCO_: carbon monoxide diffusing capacity. TSS: total severity score.

**Supplemental Table S3.** KEGG analysis for the miRNA signature associated with D_LCO_.

| **KEGG pathway** | **p-value** | **Number of Genes** |
| --- | --- | --- |
| **Viral carcinogenesis** | 3.76 X 10^-12^ | 87 |
| **Proteoglycans in cancer** | 4.03  X 10^-11^ | 84 |
| **ECM-receptor interaction** | 6.96 X 10^-11^ | 30 |
| **Prion diseases** | 4.34 X 10^-9^ | 11 |
| **Chronic myeloid leukemia** | 6.78 X 10^-9^ | 45 |
| **Hepatitis B** | 6.78 X 10^-9^ | 71 |
| **Glioma** | 1.34 X 10^-8^ | 36 |
| **Bacterial invasion of epithelial cells** | 2.04 X 10^-8^ | 46 |
| **TGF-beta signaling pathway** | 2.75 X 10^-8^ | 42 |
| **AMPK signaling pathway** | 4.14 X 10^-8^ | 66 |
| **Hippo signaling pathway** | 4.17 X 10^-8^ | 62 |
| **Pancreatic cancer** | 1.72 X 10^-7^ | 37 |
| **Neurotrophin signaling pathway** | 5.26 X 10^-7^ | 62 |
| **Fatty acid biosynthesis** | 6.11 X 10^-7^ | 4 |
| **Thyroid hormone signaling pathway** | 8.40 X 10^-7^ | 58 |
| **Renal cell carcinoma** | 1.00 X 10^-6^ | 39 |
| **FoxO signaling pathway** | 1.05 X 10^-6^ | 67 |
| **Pathways in cancer** | 8.14 X 10^-6^ | 149 |
| **Prostate cancer** | 8.14 X 10^-6^ | 48 |
| **Focal adhesion** | 1.05 X 10^-5^ | 91 |
| **Prolactin signaling pathway** | 1.22 X 10^-5^ | 37 |
| **Sphingolipid signaling pathway** | 1.40 X 10^-5^ | 52 |
| **mTOR signaling pathway** | 1.94 X 10^-5^ | 35 |
| **Small cell lung cancer** | 1.94 X 10^-5^ | 45 |
| **Estrogen signaling pathway** | 2.20 X 10^-5^ | 45 |
| **Non-small cell lung cancer** | 2.43 X 10^-5^ | 29 |
| **Signaling pathways regulating pluripotency of stem cells** | 4.82 X 10^-5^ | 59 |
| **Colorectal cancer** | 8.49 X 10^-5^ | 34 |
| **Cell cycle** | 9.35 X 10^-5^ | 55 |
| **Oocyte meiosis** | 9.75 X 10^-5^ | 50 |
| **Endometrial cancer** | 1.00 X 10^-4^ | 29 |
| **Progesterone-mediated oocyte maturation** | 1.25 X 10^-4^ | 45 |
| **Central carbon metabolism in cancer** | 6.28 X 10^-4^ | 31 |
| **Adherens junction** | 7.02 X 10^-4^ | 35 |
| **mRNA surveillance pathway** | 7.02 X 10^-4^ | 43 |
| **Insulin signaling pathway** | 7.53 X 10^-4^ | 61 |
| **Thyroid cancer** | 1.07 X 10^-3^ | 16 |
| **ErbB signaling pathway** | 1.42 X 10^-3^ | 42 |
| **Transcriptional misregulation in cancer** | 1.57 X 10^-3^ | 66 |
| **Bladder cancer** | 1.59 X 10^-3^ | 23 |
| **Toxoplasmosis** | 1.59 X 10^-3^ | 51 |
| **Ubiquitin mediated proteolysis** | 1.62 X 10^-3^ | 59 |
| **HIF-1 signaling pathway** | 1.87 X 10^-3^ | 48 |
| **Adrenergic signaling in cardiomyocytes** | 2.09 X 10^-3^ | 52 |
| **PI3K-Akt signaling pathway** | 2.21 X 10^-3^ | 119 |
| **Shigellosis** | 2.49 X 10^-3^ | 30 |
| **Lysine degradation** | 2.65 X 10^-3^ | 18 |
| **Melanoma** | 3.49 X 10^-3^ | 31 |
| **Hepatitis C** | 3.87 X 10^-3^ | 54 |
| **Protein processing in endoplasmic reticulum** | 4.21 X 10^-3^ | 65 |
| **Mucin type O-Glycan biosynthesis** | 5.99 X 10^-3^ | 12 |
| **HTLV-I infection** | 6.25 X 10^-3^ | 95 |
| **Axon guidance** | 6.43 X 10^-3^ | 48 |
| **p53 signaling pathway** | 6.55 X 10^-3^ | 32 |
| **Leukocyte transendothelial migration** | 8.28 X 10^-3^ | 44 |
| **TNF signaling pathway** | 9.05 X 10^-3^ | 46 |
| **Acute myeloid leukemia** | 1.48 X 10^-2^ | 27 |
| **Chagas disease (American trypanosomiasis)** | 1.58 X 10^-2^ | 43 |
| **Epithelial cell signaling in Helicobacter pylori infection** | 3.22 X 10^-2^ | 29 |
| **Choline metabolism in cancer** | 3.99 X 10^-2^ | 41 |
| **Platelet activation** | 4.64 X 10^-2^ | 45 |

**Supplemental Table S4.** GO analysis for the miRNA signature associated with D_LCO_.

| **GO category** | **p-value** | **Number of Genes** |
| --- | --- | --- |
| **Cellular nitrogen compound metabolic process** | 3.03 X 10^-157^ | 1325 |
| **Biosynthetic process** | 1.60 X 10^-111^ | 1106 |
| **Cellular protein modification process** | 1.31 X 10^-97^ | 724 |
| **Gene expression** | 2.79 X 10^-92^ | 261 |
| **Symbiosis. encompassing mutualism through parasitism** | 1.82 X 10^-69^ | 224 |
| **Viral process** | 1.90 X 10^-62^ | 197 |
| **Biological process** | 3.71 X 10-^53^ | 3488 |
| **Response to stress** | 3.16 X 10^-45^ | 606 |
| **Catabolic process** | 3.38 X 10^-45^ | 520 |
| **Neurotrophin trk receptor signaling pathway** | 1.42 X 10^-40^ | 108 |
| **Mitotic cell cycle** | 1.71 X 10^-39^ | 148 |
| **Cellular protein metabolic process** | 1.50 X 10^-37^ | 159 |
| **Membrane organization** | 3.30 X 10^-33^ | 194 |
| **Small molecule metabolic process** | 5.96 X 10^-32^ | 545 |
| **Cellular component assembly** | 5.73 X 10^-30^ | 347 |
| **Blood coagulation** | 1.72 X 10^-28^ | 147 |
| **Nucleobase-containing compound catabolic process** | 2.07 X 10^-28^ | 255 |
| **Cell death** | 6.37 X 10^-28^ | 266 |
| **Macromolecular complex assembly** | 3.36 X 10^-26^ | 247 |
| **Fc-epsilon receptor signaling pathway** | 4.11 X 10^-25^ | 65 |
| **Transcription. DNA-templated** | 1.12 X 10^-20^ | 610 |
| **Trif-dependent toll-like receptor signaling pathway** | 2.13 X 10^-19^ | 38 |
| **Toll-like receptor 10 signaling pathway** | 2.82 X 10^-19^ | 35 |
| **Toll-like receptor tlr1:tlr2 signaling pathway** | 4.50 X 10^-19^ | 36 |
| **Toll-like receptor tlr6:tlr2 signaling pathway** | 4.50 X 10^-19^ | 36 |
| **mRNA metabolic process** | 1.64 X 10^-18^ | 74 |
| **Toll-like receptor 9 signaling pathway** | 4.05 X 10^-18^ | 38 |
| **Protein complex assembly** | 4.05 X 10^-18^ | 205 |
| **Myd88-independent toll-like receptor signaling pathway** | 2.71 X 10^-17^ | 38 |
| **Immune system process** | 2.73 X 10^-17^ | 377 |
| **Epidermal growth factor receptor signaling pathway** | 6.26 X 10^-17^ | 75 |
| **Toll-like receptor 5 signaling pathway** | 6.26 X 10^-17^ | 35 |
| **Stress-activated mapk cascade** | 1.68 X 10^-16^ | 33 |
| **RNA metabolic process** | 6.92 X 10^-16^ | 79 |
| **Toll-like receptor 3 signaling pathway** | 7.08 X 10^-16^ | 39 |
| **DNA metabolic process** | 2.25 X 10^-15^ | 201 |
| **Fc-gamma receptor signaling pathway involved in phagocytosis** | 2.25 X 10^-15^ | 34 |
| **Chromatin organization** | 6.33 X 10^-15^ | 55 |
| **Post-translational protein modification** | 6.80 X 10^-15^ | 56 |
| **Innate immune response** | 2.63 X 10^-14^ | 190 |
| **Transcription initiation from RNA polymerase ii promoter** | 4.06 X 10^-14^ | 78 |
| **Toll-like receptor 2 signaling pathway** | 5.17 X 10^-14^ | 36 |
| **Activation of signaling protein activity involved in unfolded protein response** | 9.37 X 10^-14^ | 32 |
| **Toll-like receptor 4 signaling pathway** | 2.40 X 10^-13^ | 41 |
| **Toll-like receptor signaling pathway** | 4.58 X 10^-13^ | 45 |
| **Fibroblast growth factor receptor signaling pathway** | 1.35 X 10-^12^ | 66 |
| **Platelet activation** | 2.17 X 10^-12^ | 64 |
| **G2/m transition of mitotic cell cycle** | 2.17 X 10^-12^ | 56 |
| **Insulin receptor signaling pathway** | 3.77 X 10^-12^ | 63 |
| **Transforming growth factor beta receptor signaling pathway** | 8.84 X 10^-11^ | 69 |
| **Myd88-dependent toll-like receptor signaling pathway** | 1.11 X 10^-10^ | 38 |
| **Cellular lipid metabolic process** | 2.00 X 10^-10^ | 46 |
| **Cytoskeleton organization** | 7.74 X 10^-10^ | 170 |
| **Phosphatidylinositol-mediated signaling** | 8.98 X 10^-10^ | 48 |
| **Intrinsic apoptotic signaling pathway** | 1.92 X 10^-8^ | 30 |
| **Cell junction organization** | 2.14 X 10^-8^ | 49 |
| **Cellular component disassembly involved in execution phase of apoptosis** | 3.35 X 10^-8^ | 20 |
| **Vesicle-mediated transport** | 5.27 X 10^-8^ | 236 |
| **Protein n-linked glycosylation via asparagine** | 8.49 X 10^-8^ | 34 |
| **Extracellular matrix disassembly** | 1.73 X 10^-7^ | 35 |
| **Platelet degranulation** | 3.06 X 10^-7^ | 26 |
| **Cell cycle** | 3.42 X 10^-7^ | 222 |
| **Apoptotic signaling pathway** | 3.58 X 10^-7^ | 44 |
| **Positive regulation of protein insertion into mitochondrial membrane involved in apoptotic signaling pathway** | 3.58 X 10^-7^ | 16 |
| **mRNA processing** | 3.90 X 10^-7^ | 138 |
| **Termination of RNA polymerase II transcription** | 3.92 X 10^-7^ | 20 |
| **Viral life cycle** | 7.23 X 10^-7^ | 33 |
| **Mitotic nuclear envelope disassembly** | 1.29 X 10^-6^ | 16 |
| **Nucleocytoplasmic transport** | 1.31 X 10^-6^ | 94 |
| **mRNA 3'-end processing** | 2.11 X 10^-6^ | 18 |
| **Cell proliferation** | 2.88 X 10^-6^ | 156 |
| **Transcription from RNA polymerase II promoter** | 3.21 X 10^-6^ | 154 |
| **RNA splicing** | 4.37 X 10^-6^ | 88 |
| **Axon guidance** | 7.56 X 10^-6^ | 112 |
| **Nuclear-transcribed mRNA catabolic process. deadenylation-dependent decay** | 7.71 X 10^-6^ | 23 |
| **Endoplasmic reticulum unfolded protein response** | 1.01 X 10^-5^ | 42 |
| **Extracellular matrix organization** | 1.08 X 10^-5^ | 90 |
| **Negative regulation of transcription from RNA polymerase ii promoter** | 1.08 X 10^-5^ | 247 |
| **Type I interferon signaling pathway** | 1.11 X 10^-5^ | 26 |
| **Jak-stat cascade involved in growth hormone signaling pathway** | 2.19 X 10^-5^ | 13 |
| **Cell cycle arrest** | 2.29 X 10^-5^ | 56 |
| **Cell junction assembly** | 2.36 X 10^-5^ | 21 |
| **Nucleotide-binding domain. leucine rich repeat containing receptor signaling pathway** | 3.51 X 10^-5^ | 18 |
| **Protein targeting** | 4.40 X 10^-5^ | 72 |
| **Hexose transport** | 4.69 X 10^-5^ | 15 |
| **Antigen processing and presentation of exogenous peptide antigen via mhc class ii** | 5.53 X 10^-5^ | 35 |
| **Regulation of transcription from RNA polymerase II promoter in response to hypoxia** | 5.84 X 10^-5^ | 14 |
| **G1/S transition of mitotic cell cycle** | 6.59 X 10^-5^ | 50 |
| **Cellular component movement** | 6.69 X 10^-5^ | 33 |
| **Chromatin modification** | 1.08 X 10^-4^ | 56 |
| **Phosphatidylinositol biosynthetic process** | 1.08 X 10^-4^ | 22 |
| **Generation of precursor metabolites and energy** | 1.31 X 10^-4^ | 74 |
| **Cytokine-mediated signaling pathway** | 1.72 X 10^-4^ | 76 |
| **Leukocyte migration** | 1.78 X 10^-4^ | 32 |
| **Regulation of glucose transport** | 1.79 X 10^-4^ | 13 |
| **Negative regulation of cell proliferation** | 1.81 X 10^-4^ | 142 |
| **Nuclear-transcribed mRNA catabolic process. nonsense-mediated decay** | 2.54 X 10^-4^ | 37 |
| **Positive regulation of type I interferon production** | 2.60 X 10^-4^ | 21 |
| **Positive regulation of apoptotic process** | 5.29 X 10^-4^ | 113 |
| **Nucleotide-binding oligomerization domain containing signaling pathway** | 5.41 X 10^-4^ | 12 |
| **Positive regulation of transcription. DNA-templated** | 7.04 X 10^-4^ | 207 |
| **Post-Golgi vesicle-mediated transport** | 7.79 X 10^-4^ | 17 |
| **Regulation of ubiquitin-protein ligase activity involved in mitotic cell cycle** | 8.84 X 10^-4^ | 20 |
| **Cell motility** | 9.25 X 10^-4^ | 118 |
| **Phospholipid metabolic process** | 9.63 X 10^-4^ | 41 |
| **Notch signaling pathway** | 9.85 X 10^-4^ | 54 |
| **Protein polyubiquitination** | 1.17 X 10^-3^ | 45 |
| **mRNA splicing. via spliceosome** | 1.29 X 10^-3^ | 63 |
| **Protein maturation** | 1.41 X 10^-3^ | 49 |
| **Anatomical structure morphogenesis** | 1.79 X 10^-3^ | 29 |
| **Glycosaminoglycan metabolic process** | 1.94 X 10^-3^ | 25 |
| **Protein ubiquitination** | 1.94 X 10^-3^ | 130 |
| **Viral transcription** | 3.04 X 10^-3^ | 20 |
| **Ribonucleoprotein complex assembly** | 3.04 X 10^-3^ | 37 |
| **In utero embryonic development** | 3.16 X 10^-3^ | 85 |
| **Vitamin metabolic process** | 3.47 X 10^-3^ | 19 |
| **Ras protein signal transduction** | 3.96 X 10^-3^ | 31 |
| **Nuclear-transcribed mRNA poly(A) tail shortening** | 4.20 X 10^-3^ | 14 |
| **Srp-dependent cotranslational protein targeting to membrane** | 4.36 X 10^-3^ | 30 |
| **Cellular response to hypoxia** | 4.59 X 10^-3^ | 42 |
| **Positive regulation of ubiquitin-protein ligase activity involved in mitotic cell cycle** | 5.13 X 10^-3^ | 18 |
| **Positive regulation of viral transcription** | 5.44 X 10^-3^ | 15 |
| **Apoptotic process** | 5.44 X 10^-3^ | 195 |
| **Regulation of small GTPase mediated signal transduction** | 5.44 X 10^-3^ | 50 |
| **Transcription elongation from RNA polymerase II promoter** | 5.44 X 10^-3^ | 22 |
| **Inositol phosphate metabolic process** | 5.94 X 10^-3^ | 13 |
| **Positive regulation of muscle cell differentiation** | 5.94 X 10^-3^ | 12 |
| **Regulation of transcription involved in g1/s transition of mitotic cell cycle** | 5.94 X 10^-3^ | 12 |
| **O-glycan processing** | 6.07 X 10^-3^ | 15 |
| **Water-soluble vitamin metabolic process** | 6.37 X 10^-3^ | 17 |
| **Negative regulation of transforming growth factor beta receptor signaling pathway** | 7.67 X 10^-3^ | 31 |
| **Energy reserve metabolic process** | 1.10 X 10^-2^ | 23 |
| **Translational termination** | 1.10 X 10^-2^ | 23 |
| **Nitric oxide metabolic process** | 1.11 X 10^-2^ | 9 |
| **Cofactor metabolic process** | 1.12 X 10^-2^ | 52 |
| **Negative regulation of apoptotic process** | 1.24 X 10^-2^ | 159 |
| **Signal transduction** | 1.33 X 10^-2^ | 989 |
| **Cellular response to ionizing radiation** | 1.46 X 10^-2^ | 18 |
| **Negative regulation of transcription. DNA-templated** | 1.57 X 10^-2^ | 174 |
| **Glycerophospholipid biosynthetic process** | 1.72 X 10^-2^ | 20 |
| **Regulation of defense response to virus by virus** | 2.26 X 10^-2^ | 9 |
| **Response to virus** | 2.47 X 10^-2^ | 45 |
| **Androgen receptor signaling pathway** | 2.51 X 10^-2^ | 18 |
| **Long-chain fatty-acyl-coa biosynthetic process** | 2.57 X 10^-2^ | 7 |
| **Regulation of nitric-oxide synthase activity** | 2.77 X 10^-2^ | 9 |
| **mRNA export from nucleus** | 2.96 X 10^-2^ | 24 |
| **Transport** | 3.05 X 10^-2^ | 757 |
| **Muscle cell differentiation** | 3.41 X 10^-2^ | 15 |
| **Anaphase-promoting complex-dependent proteasomal ubiquitin-dependent protein catabolic process** | 3.61 X 10^-2^ | 20 |
| **Negative regulation of cell migration** | 3.91 X 10^-2^ | 38 |
| **Collagen catabolic process** | 3.91 X 10^-2^ | 20 |

**Supplemental Table S5.** KEGG analysis for the miRNA signature associated with TSS.

| **KEGG pathway** | **p-value** | **Number of Genes** |
| --- | --- | --- |
| **Fatty acid biosynthesis** | 1.29E X 10^-15^ | 6 |
| **Proteoglycans in cancer** | 1.53 X 10^-10^ | 80 |
| **Hippo signaling pathway** | 2.75 X 10^-7^ | 57 |
| **Fatty acid metabolism** | 2.75 X 10^-7^ | 16 |
| **Hepatitis B** | 4.08 X 10^-6^ | 55 |
| **Cell cycle** | 1.49 X 10^-5^ | 55 |
| **Adherens junction** | 2.92 X 10^-5^ | 32 |
| **Pathways in cancer** | 2.92 X 10^-5^ | 132 |
| **Viral carcinogenesis** | 1.68 X 10^-4^ | 65 |
| **Endocytosis** | 1.72 X 10^-4^ | 75 |
| **Chronic myeloid leukemia** | 5.45 X 10^-4^ | 32 |
| **Other types of O-glycan biosynthesis** | 5.47 X 10^-4^ | 10 |
| **Colorectal cancer** | 5.61 X 10^-4^ | 27 |
| **ECM-receptor interaction** | 5.61 X 10^-4^ | 26 |
| **Neurotrophin signaling pathway** | 2.79 X 10^-3^ | 47 |
| **Glycosaminoglycan biosynthesis - keratan sulfate** | 3.07 X 10^-3^ | 6 |
| **Bacterial invasion of epithelial cells** | 3.38 X 10^-3^ | 33 |
| **FoxO signaling pathway** | 3.38 X 10^-3^ | 50 |
| **Thyroid hormone signaling pathway** | 3.38 X 10^-3^ | 44 |
| **p53 signaling pathway** | 3.44 X 10^-3^ | 32 |
| **Fatty acid elongation** | 3.44 X 10^-3^ | 8 |
| **Focal adhesion** | 3.44 X 10^-3^ | 74 |
| **Renal cell carcinoma** | 3.79 X 10^-3^ | 27 |
| **Small cell lung cancer** | 3.79 X 10^-3^ | 36 |
| **TGF-beta signaling pathway** | 3.94 X 10^-3^ | 29 |
| **HTLV-I infection** | 4.76 X 10^-3^ | 86 |
| **Transcriptional misregulation in cancer** | 4.88 X 10^-3^ | 62 |
| **Glioma** | 5.78 X 10^-3^ | 25 |
| **Pancreatic cancer** | 5.78 X 10^-3^ | 29 |
| **Lysine degradation** | 8.17 X 10^-3^ | 18 |
| **Bladder cancer** | 1.06 X 10^-2^ | 19 |
| **Epstein-Barr virus infection** | 1.35 X 10^-2^ | 71 |
| **RNA transport** | 1.52 X 10^-2^ | 57 |
| **Chagas disease (American trypanosomiasis)** | 2.25 X 10^-2^ | 37 |
| **Vitamin B6 metabolism** | 2.70 X 10^-2^ | 3 |
| **TNF signaling pathway** | 3.40 X 10^-2^ | 38 |
| **Sphingolipid signaling pathway** | 3.85 X 10^-2^ | 42 |
| **Protein processing in endoplasmic reticulum** | 3.91 X 10^-2^ | 57 |
| **Non-small cell lung cancer** | 3.97 X 10^-2^ | 23 |
| **Apoptosis** | 4.26 X 10^-2^ | 30 |
| **HIF-1 signaling pathway** | 4.61 X 10^-2^ | 38 |
| **Arrhythmogenic right ventricular cardiomyopathy (ARVC)** | 4.82 X 10^-2^ | 21 |

**Supplemental Table S6.** GO analysis for the miRNA signature associated with TSS.

| **GO category** | **p-value** | **Number of Genes** |
| --- | --- | --- |
| **Cellular nitrogen compound metabolic process** | 5.22 X 10^-142^ | 1242 |
| **Biosynthetic process** | 3.95 X 10^-94^ | 1020 |
| **Gene expression** | 5.06 X 10^-80^ | 239 |
| **Cellular protein modification process** | 1.07 X 10^-77^ | 653 |
| **Viral process** | 3.64 X 10^-63^ | 193 |
| **Symbiosis** | 3.09 X 10^-62^ | 208 |
| **Small molecule metabolic process** | 4.59 X 10^-50^ | 582 |
| **Catabolic process** | 1.36 X 10^-49^ | 513 |
| **Biological process** | 2.93 X 10^-41^ | 3276 |
| **Cellular protein metabolic process** | 8.40 X 10^-38^ | 155 |
| **Membrane organization** | 9.19 X 10^-36^ | 193 |
| **Cellular component assembly** | 1.83 X 10^-34^ | 347 |
| **Macromolecular complex assembly** | 1.83 X 10^-34^ | 259 |
| **Mitotic cell cycle** | 3.38 X 10^-33^ | 134 |
| **Neurotrophin TRK receptor signaling pathway** | 1.32 X 10^-31^ | 94 |
| **Nucleobase-containing compound catabolic process** | 3.51 X 10^-31^ | 253 |
| **Response to stress** | 5.97 X 10^-31^ | 534 |
| **Cell death** | 1.19 X 10^-29^ | 261 |
| **Protein complex assembly** | 1.44 X 10^-25^ | 218 |
| **Blood coagulation** | 4.33 X 10^-22^ | 130 |
| **mRNA metabolic process** | 2.13 X 10^-20^ | 75 |
| **Fc-epsilon receptor signaling pathway** | 7.96 X 10^-20^ | 57 |
| **RNA metabolic process** | 4.87 X 10^-17^ | 79 |
| **DNA metabolic process** | 2.46 X 10^-16^ | 197 |
| **Epidermal growth factor receptor signaling pathway** | 3.70 X 10^-15^ | 70 |
| **Cellular component disassembly involved in execution phase of apoptosis** | 1.79 X 10^-14^ | 26 |
| **Post-translational protein modification** | 2.09 X 10^-14^ | 54 |
| **Immune system process** | 2.55 X 10^-14^ | 349 |
| **Chromatin organization** | 8.67 X 10^-14^ | 52 |
| **Cellular lipid metabolic process** | 7.05 X 10^-13^ | 49 |
| **Mitotic nuclear envelope disassembly** | 7.17 X 10^-12^ | 21 |
| **Toll-like receptor TLR1:TLR2 signaling pathway** | 7.97 X 10^-12^ | 28 |
| **Toll-like receptor TLR6:TLR2 signaling pathway** | 7.97 X 10^-12^ | 28 |
| **Toll-like receptor 10 signaling pathway** | 8.23 X 10^-12^ | 27 |
| **Extracellular matrix organization** | 4.15 X 10^-11^ | 104 |
| **Cell motility** | 6.16 X 10^-11^ | 143 |
| **TRIF-dependent toll-like receptor signaling pathway** | 9.95 X 10^-11^ | 28 |
| **Cell junction organization** | 1.28 X 10^-10^ | 52 |
| **Extracellular matrix disassembly** | 2.07 X 10^-10^ | 39 |
| **Transcription initiation from RNA polymerase II promoter** | 2.28 X 10^-10^ | 68 |
| **Toll-like receptor 5 signaling pathway** | 3.12 X 10^-10^ | 27 |
| **Vesicle-mediated transport** | 3.12 X 10^-10^ | 237 |
| **Cytoskeleton organization** | 6.17 X 10^-10^ | 164 |
| **Toll-like receptor 9 signaling pathway** | 6.17 X 10^-10^ | 28 |
| **Platelet degranulation** | 8.25 X 10^-10^ | 29 |
| **Transforming growth factor beta receptor signaling pathway** | 1.92 X 10^-9^ | 64 |
| **Myd88-independent toll-like receptor signaling pathway** | 1.95 X 10^-9^ | 28 |
| **Toll-like receptor 4 signaling pathway** | 4.10 X 10^-9^ | 34 |
| **Toll-like receptor 2 signaling pathway** | 4.11 X 10^-9^ | 29 |
| **Cellular component movement** | 6.77 X 10^-9^ | 40 |
| **G2/M transition of mitotic cell cycle** | 7.22 X 10^-9^ | 48 |
| **Platelet activation** | 8.02 X 10^-9^ | 55 |
| **Toll-like receptor 3 signaling pathway** | 1.08 X 10^-8^ | 29 |
| **Toll-like receptor signaling pathway** | 1.15 X 10^-8^ | 37 |
| **Viral life cycle** | 1.53 X 10^-8^ | 35 |
| **Protein N-linked glycosylation via asparagine** | 2.34 X 10^-8^ | 34 |
| **Protein targeting** | 3.64 X 10^-8^ | 79 |
| **Transcription. DNA-templated** | 4.08 X 10^-8^ | 510 |
| **Regulation of transcription from RNA polymerase II promoter in response to hypoxia** | 8.99 X 10^-8^ | 15 |
| **Fibroblast growth factor receptor signaling pathway** | 2.41 X 10^-7^ | 53 |
| **Glycosaminoglycan metabolic process** | 2.93 X 10^-7^ | 32 |
| **Apoptotic signaling pathway** | 2.23 X 10^-6^ | 41 |
| **Activation of signaling protein activity involved in unfolded protein response** | 2.72 X 10^-6^ | 22 |
| **Nuclear-transcribed mRNA catabolic process deadenylation-dependent decay** | 3.58 X 10^-6^ | 23 |
| **Hexose transport** | 4.63 X 10^-6^ | 16 |
| **Innate immune response** | 7.79 X 10^-6^ | 150 |
| **Myd88-dependent toll-like receptor signaling pathway** | 8.34 X 10^-6^ | 29 |
| **Positive regulation of type I interferon production** | 9.74 X 10^-6^ | 23 |
| **Regulation of ubiquitin-protein ligase activity involved in mitotic cell cycle** | 9.74 X 10^-6^ | 23 |
| **Cell junction assembly** | 1.19 X 10^-5^ | 21 |
| **Cytoskeleton-dependent intracellular transport** | 1.28 X 10^-5^ | 34 |
| **Phosphatidylinositol-mediated signaling** | 1.54 X 10^-5^ | 38 |
| **G1/S transition of mitotic cell cycle** | 1.71 X 10^-5^ | 50 |
| **Regulation of glucose transport** | 1.78 X 10^-5^ | 14 |
| **Positive regulation of ubiquitin-protein ligase activity involved in mitotic cell cycle** | 2.09 X 10^-5^ | 22 |
| **Insulin receptor signaling pathway** | 2.82 X 10^-5^ | 46 |
| **Protein maturation** | 4.95 X 10^-5^ | 52 |
| **SRP-dependent cotranslational protein targeting to membrane** | 5.29 X 10^-5^ | 34 |
| **Axon guidance** | 5.29 X 10^-5^ | 104 |
| **Fc-gamma receptor signaling pathway involved in phagocytosis** | 5.84 X 10^-5^ | 20 |
| **Cell proliferation** | 6.07 X 10^-5^ | 143 |
| **Sulfur compound metabolic process** | 6.48 X 10^-5^ | 64 |
| **Leukocyte migration** | 6.66 X 10^-5^ | 32 |
| **Cell cycle** | 7.61 X 10^-5^ | 199 |
| **RNA splicing** | 7.82 X 10^-5^ | 80 |
| **Nucleobase-containing small molecule metabolic process** | 8.11 X 10^-5^ | 20 |
| **Regulation of defense response to virus by virus** | 9.61 X 10^-5^ | 12 |
| **Post-Golgi vesicle-mediated transport** | 1.02 X 10^-4^ | 18 |
| **Sphingolipid biosynthetic process** | 1.02 X 10^-4^ | 18 |
| **Cell cycle arrest** | 1.03 X 10^-4^ | 52 |
| **DNA damage response. signal transduction by p53 class mediator resulting in cell cycle arrest** | 1.15 X 10^-4^ | 23 |
| **Stress-activated MAPK cascade** | 1.22 X 10^-4^ | 18 |
| **Termination of RNA polymerase II transcription** | 1.44 X 10^-4^ | 16 |
| **Anaphase-promoting complex-dependent proteasomal ubiquitin-dependent protein catabolic process** | 1.51 X 10^-4^ | 25 |
| **Nuclear-transcribed mRNA catabolic process. nonsense-mediated decay** | 2.01 X 10^-4^ | 36 |
| **Nucleocytoplasmic transport** | 2.01 X 10^-4^ | 82 |
| **Ribonucleoprotein complex assembly** | 2.15 X 10^-4^ | 39 |
| **Positive regulation of apoptotic process** | 2.27 X 10^-4^ | 110 |
| **Phospholipid metabolic process** | 2.97 X 10^-4^ | 41 |
| **mRNA processing** | 3.02 X 10^-4^ | 119 |
| **Antigen processing and presentation of exogenous peptide antigen via MHC class II** | 3.08 X 10^-4^ | 32 |
| **In utero embryonic development** | 3.30 X 10^-4^ | 86 |
| **Transcription from RNA polymerase II promoter** | 3.33 X 10^-4^ | 137 |
| **Viral transcription** | 4.82 X 10^-4^ | 21 |
| **mRNA 3'-end processing** | 7.57 X 10^-4^ | 14 |
| **Cellular response to hypoxia** | 7.61 X 10^-4^ | 43 |
| **DNA strand elongation involved in DNA replication** | 7.70 X 10^-4^ | 12 |
| **Positive regulation of transcription. DNA-templated** | 8.23 X 10^-4^ | 197 |
| **Cytokine-mediated signaling pathway** | 8.95 X 10^-4^ | 70 |
| **Notch signaling pathway** | 8.95 X 10^-4^ | 52 |
| **Homeostatic process** | 1.27 X 10^-3^ | 156 |
| **Negative regulation of apoptotic process** | 1.28 X 10^-3^ | 159 |
| **Glycerophospholipid biosynthetic process** | 1.32 X 10^-3^ | 22 |
| **Negative regulation of epidermal growth factor receptor signaling pathway** | 1.53 X 10^-3^ | 18 |
| **Phosphatidylinositol biosynthetic process** | 1.74 X 10^-3^ | 19 |
| **Lipid metabolic process** | 1.97 X 10^-3^ | 235 |
| **Translational termination** | 2.20 X 10^-3^ | 24 |
| **Response to unfolded protein** | 2.44 X 10^-3^ | 24 |
| **mRNA splicing. via spliceosome** | 2.87 X 10^-3^ | 59 |
| **Long-chain fatty-acyl-coa biosynthetic process** | 2.89 X 10^-3^ | 8 |
| **Platelet-derived growth factor receptor signaling pathway** | 3.24 X 10^-3^ | 21 |
| **Ras protein signal transduction** | 3.46 X 10^-3^ | 30 |
| **Activation of MAPKK activity** | 3.78 X 10^-3^ | 26 |
| **Positive regulation of muscle cell differentiation** | 3.78 X 10^-3^ | 12 |
| **Negative regulation of transcription from RNA polymerase II promoter** | 4.10 X 10^-3^ | 217 |
| **Protein polyubiquitination** | 5.23 X 10^-3^ | 41 |
| **Cofactor metabolic process** | 6.21 X 10^-3^ | 51 |
| **Activation of JUN kinase activity** | 6.78 X 10^-3^ | 19 |
| **Negative regulation of transforming growth factor beta receptor signaling pathway** | 7.01 X 10^-3^ | 30 |
| **Apoptotic process** | 7.20 X 10^-3^ | 185 |
| **Muscle cell differentiation** | 7.25 X 10^-3^ | 16 |
| **Intrinsic apoptotic signaling pathway** | 7.42 X 10^-3^ | 19 |
| **Collagen catabolic process** | 9.19 X 10^-3^ | 21 |
| **Generation of precursor metabolites and energy** | 9.77 X 10^-3^ | 63 |
| **Endoplasmic reticulum unfolded protein response** | 1.13 X 10^-2^ | 32 |
| **Transport** | 1.28 X 10^-2^ | 726 |
| **Adherens junction organization** | 1.32 X 10^-2^ | 14 |
| **Dolichol-linked oligosaccharide biosynthetic process** | 1.32 X 10^-2^ | 12 |
| **Inositol phosphate metabolic process** | 1.32 X 10^-2^ | 12 |
| **Negative regulation of cell proliferation** | 1.38 X 10^-2^ | 124 |
| **Positive regulation of I-kappab kinase/NF-kappab signaling** | 1.74 X 10^-2^ | 60 |
| **Notch receptor processing** | 1.81 X 10^-2^ | 12 |
| **Sulfur amino acid metabolic process** | 1.95 X 10^-2^ | 9 |
| **Triglyceride biosynthetic process** | 2.08 X 10^-2^ | 15 |
| **Peptidyl-threonine phosphorylation** | 2.55 X 10^-2^ | 22 |
| **Regulation of small GTPase mediated signal transduction** | 2.77 X 10^-2^ | 45 |
| **Cell-cell junction organization** | 3.12 X 10^-2^ | 20 |
| **Protein stabilization** | 3.45 X 10^-2^ | 40 |
| **Nitric oxide metabolic process** | 3.70 X 10^-2^ | 8 |
| **Erythrocyte differentiation** | 3.73 X 10^-2^ | 24 |
| **Cellular amino acid metabolic process** | 4.21 X 10^-2^ | 80 |
| **Positive regulation of protein insertion into mitochondrial membrane involved in apoptotic signaling pathway** | 4.25 X 10^-2^ | 9 |
